# Aerobic Microbial Respiration In Oceanic Oxygen Minimum Zones

**DOI:** 10.1371/journal.pone.0133526

**Published:** 2015-07-20

**Authors:** Tim Kalvelage, Gaute Lavik, Marlene M. Jensen, Niels Peter Revsbech, Carolin Löscher, Harald Schunck, Dhwani K. Desai, Helena Hauss, Rainer Kiko, Moritz Holtappels, Julie LaRoche, Ruth A. Schmitz, Michelle I. Graco, Marcel M. M. Kuypers

**Affiliations:** 1 Biogeochemistry Department, Max Planck Institute for Marine Microbiology, Bremen, Germany; 2 Department of Biological Sciences, University of Aarhus, Aarhus, Denmark; 3 Institute for General Microbiology, Christian Albrechts University Kiel, Kiel, Germany; 4 GEOMAR Helmholtz Centre for Ocean Research Kiel, Kiel, Germany; 5 Dirección de Investigaciones Oceanográficas, Instituto del Mar del Perú, Lima, Peru; Fudan University, CHINA

## Abstract

Oxygen minimum zones are major sites of fixed nitrogen loss in the ocean. Recent studies have highlighted the importance of anaerobic ammonium oxidation, anammox, in pelagic nitrogen removal. Sources of ammonium for the anammox reaction, however, remain controversial, as heterotrophic denitrification and alternative anaerobic pathways of organic matter remineralization cannot account for the ammonium requirements of reported anammox rates. Here, we explore the significance of microaerobic respiration as a source of ammonium during organic matter degradation in the oxygen-deficient waters off Namibia and Peru. Experiments with additions of double-labelled oxygen revealed high aerobic activity in the upper OMZs, likely controlled by surface organic matter export. Consistently observed oxygen consumption in samples retrieved throughout the lower OMZs hints at efficient exploitation of vertically and laterally advected, oxygenated waters in this zone by aerobic microorganisms. In accordance, metagenomic and metatranscriptomic analyses identified genes encoding for aerobic terminal oxidases and demonstrated their expression by diverse microbial communities, even in virtually anoxic waters. Our results suggest that microaerobic respiration is a major mode of organic matter remineralization and source of ammonium (~45-100%) in the upper oxygen minimum zones, and reconcile hitherto observed mismatches between ammonium producing and consuming processes therein.

## Introduction

Most of the organic matter in the world’s oceans is remineralized via aerobic respiration by heterotrophic microorganisms. Only when oxygen (O_2_) becomes scarce, microorganisms use thermodynamically less favourable electron acceptors, predominantly nitrate (NO_3_^-^), for the oxidation of organic matter [1]. Large, permanently O_2_-depleted water masses favouring NO_3_^-^ respiration, so-called oxygen minimum zones (OMZs), are found in association with tropical and subtropical upwelling systems [2]. These regions are characterized by high surface productivity and thus strong O_2_ depletion via degradation of sinking organic matter at mid-depth, exacerbated by limited O_2_ replenishment [3]. Nitrate respiration in OMZs accounts for ~20–40% of global oceanic nitrogen (N) loss [4]. The N-deficient OMZ waters (relative to phosphorus) are eventually upwelled and result in largely N-limited surface primary production at low latitudes [5]. Hence, despite a combined volume of only ~1% (O_2_ ≤20 μmol l^-1^) of the global ocean [1], OMZs play an important role in regulating phytoplankton nutrient availability, and thus carbon (C) fixation in the oceans.

Traditionally, OMZ N-loss has been attributed to heterotrophic denitrification [6–8], the step-wise reduction of NO_3_^-^ to dinitrogen gas (N_2_) coupled to the oxidation of organic matter by facultative anaerobes at low O_2_ tensions [9]. In the last decade, however, numerous studies have identified anammox, the anaerobic oxidation of NH_4_^+^ with nitrite (NO_2_^-^), as a major N_2_-forming pathway in OMZs, often exceeding N-loss via denitrification [10–14]. Assuming denitrification to be the only N-remineralization pathway in OMZs, anammox activity therein should be constrained by the amount of NH_4_^+^ released during heterotrophic denitrification [15,16]. The apparent decoupling of the two processes requires sources of NH_4_^+^ other than denitrification. Alternative anaerobic NH_4_^+^-producing pathways in OMZs include heterotrophic NO_3_^-^ reduction to NO_2_^-^, dissimilatory NO_3_^-^ reduction to NH_4_^+^ (DNRA) and sulphate reduction coupled to organic matter degradation [13,17–19]; yet, rates reported for these processes are neither sufficient to fully account for estimated remineralization of sinking organic matter in OMZs [14] nor to explain the NH_4_^+^ demands of concurrent anammox activity. Particularly large imbalances between NH_4_^+^ sources and sinks persist at the upper OMZ boundaries, where rates of anammox as well as aerobic NH_4_^+^ oxidation often peak [14,17,20,21].

Microaerobic respiration of organic matter has been suggested to provide the “missing” NH_4_^+^ in the upper OMZs [14,17]. Owing to the detection limit of conventional O_2_ measurements, remineralization of organic matter below ~5 μmol l^-1^ of O_2_ is commonly believed to proceed via NO_3_^-^ respiration [22]. However, during a recent hydrochemical survey of the South Pacific OMZ with switchable trace amount oxygen (STOX) sensors, accumulations of NO_2_^-^, considered a proxy for active NO_3_^-^ respiration, were only observed at O_2_ levels <50 nmol l^-1^ [23]. At the same time, O_2_-dependent nitrification at O_2_ levels ≤1 μmol l^-1^ in OMZs hints at aerobic microbial respiration to be well adapted to nanomolar O_2_ concentrations [14,24,25]. In accordance, highly sensitive measurements of (microbial) O_2_ consumption in the OMZs off Chile and Mexico recently revealed apparent half-saturation coefficients (K_m_) for aerobic respiration of ~10–200 nmol O_2_ l^-1^ [26].

Highly efficient O_2_ scavenging is a prerequisite for maintaining anoxic conditions in OMZs against the transport of O_2_-bearing water masses via turbulent mixing, local downwelling or lateral advection. Fundamentally, the balance between microbial O_2_ uptake and downward O_2_ transport via turbulent diffusion results in a gradual decrease of O_2_ and the typical oxycline formation. In such a simplistic setting, sub-oxycline waters quickly become functionally anoxic [23]. Intrusions, local downwelling and lateral advection of oxygenated water masses, however, provide effective means of O_2_ transport into the OMZ interior [27,28]. To maintain anoxia in OMZs, such O_2_ injections must either be rare events or well-adapted, opportunistic microbial communities must rapidly draw down any O_2_ available.

Here, we assessed the potential of microaerobic respiration and the importance of aerobic organic matter degradation as a source of NH_4_^+^ in the OMZs off Namibia and Peru, using an ^18-18^O_2_ labelling approach suitable for O_2_ consumption measurements at low O_2_ concentrations [29]. Rate measurements were complemented by analyses of metagenomes and metatranscriptomes from the South Pacific OMZ, for presence and expression of key-functional genes involved in aerobic respiration. Further, we explored the effects of O_2_ depletion associated with marine snow particles on microbial respiration, by combining ^18-18^O_2_ labelling experiments with *in-situ* particle size analysis and modelling of aggregate-size-dependent respiration.

## Materials and Methods

### Water sampling and physico-chemical measurements

Samples were taken on cruises M76-2 and M77-3 over the Namibian shelf (May to June 2008) and in the OMZ off Peru (December 2008 to January 2009), respectively, on board R/V Meteor (Table 1). Seawater was collected with either a conductivity-temperature-depth (CTD) rosette system fitted with 10-L Niskin bottles or a pump-CTD system (depth range: ~375 m). Off Namibia, a custom-built bottom water sampler [30] was used to collect additional samples from the benthic boundary layer (BBL). Oxygen was measured with a conventional amperometric microsensor and a CTD-mounted switchable trace amount oxygen (STOX) sensor [31] (detection limit: 50–100 nmol l^-1^) for high-accuracy O_2_ measurements at selected depths. Continuous vertical profiles of chlorophyll *a* were obtained fluorometrically and calibrated against discrete values derived from acetone extraction. Ammonium concentrations were determined fluorometrically [32] on discrete high-resolution samples (1–2 m). Samples for particulate organic nitrogen (PON) were filtered onto pre-combusted GF/F filters (Whatman), stored frozen, and measured on an elemental analyzer (EURO EA and Thermo Flash EA, 1112 Series) after drying and decalcification with fuming hydrochloric acid.

**Table 1:** Overview of sampling locations and times for the various types of data considered in this study.

Cruise	OMZ	Lat/Lon	Year	Season	Data obtained
Meteor 76–2	Namibia	19–23°S/12-14°E	2008	Austral autumn	O_2_ consumption rates, N-cycling rates
MOOMZ-1 [34]	Chile	20°07’S/70°23’W	2008	Austral winter	Terminal respiratory oxidase gene and transcript abundance
Meteor 77–3	Peru	4–16°S/75-84°W	2008–2009	Austral summer	O_2_ consumption rates, N-cycling rates, terminal respiratory oxidase gene abundance
Meteor 93	Peru	12–14°S /76-79°W	2013	Austral summer	*In-situ* particle size spectra

Particle abundances and size distributions were measured during cruise M93 (February to March 2013) to the central Peruvian OMZ (Table 1), using an Underwater Vision Profiler (UVP5) [33]. A total of 138 UVP5 profiles, quantifying particles with an equivalent spherical diameter (ESD) of 0.06–26.8 mm, were obtained.

### Determination of O_2_ consumption rates

Microaerobic respiration in the Namibian as well as the coastal and offshore Peruvian OMZ was measured as the consumption of ^18-18^O_2_ in time-series incubations. At each station, up to six depths were chosen for ^18-18^O_2_ labelling experiments (S1 Table). A detailed description of the experimental procedure is given in reference [29]. Briefly, Helium-purged water samples were adjusted to the following ^18-18^O_2_ concentrations by adding a defined volume of sterile-filtered seawater containing ~1 mmol ^18-18^O_2_ l^-1^ (Sigma-Aldrich, Germany): ~2.5 μmol l^-1^ (Namibian OMZ), ~2.5–7.5 μmol l^-1^ (upper Peruvian OMZ) and ~1 μmol l^-1^ (Peruvian OMZ core). At selected stations, additional O_2_ sensitivity assays were carried out, in which replicate samples were adjusted to different ^18-18^O_2_ concentrations in the range of ~0.5–20 μmol l^-1^ (S2 Table). Following O_2_ adjustment, subsamples were filled into 12-ml Exetainers (Labco, UK), using Helium overpressure to avoid ^16-16^O_2_ contaminations. One Exetainer each was sacrificed to determine initial total O_2_ (^18-18^O_2_ + ^16-16^O_2_) concentrations with a fast-responding Clark-type O_2_ microsensor (MPI Bremen; detection limit: ~0.5 μmol l^-1^). Samples were incubated in the dark at mean *in-situ* temperatures and duplicates were inactivated after ~0, 3, 6, 12, 24 and 48 h by adding saturated mercuric chloride. Final ^18-18^O_2_ concentrations were determined using membrane inlet mass spectrometry (MIMS; GAM200, IPI) in a shore-based laboratory. A two-point calibration was performed based on the ^16-16^O_2_ reading for air-saturated water pumped across the inlet membrane and the ^18-18^O_2_-background signal with the pump turned off. Oxygen consumption rates were calculated from the slope of linear regression of ^18-18^O_2_ concentrations as a function of time and corrected for ^16-16^O_2_ background concentrations. Samples incubated for ~48 h were not considered if a non-linear decrease of ^18-18^O_2_ was observed for the final incubation period (24–48 h).

### Modelling of O_2_ consumption rates

The diffusive O_2_ flux at the upper OMZ boundary was calculated from turbulent diffusivity and the O_2_ concentration gradient according to Fick’s law. Oxygen consumption rates were then estimated from O_2_ flux gradients. A more detailed description of the modelling approach is given in the S1 File.

### Metagenomic and metatranscriptomic analyses

In the Peruvian OMZ, large-volume samples (>300 L) for nucleic acid extraction were collected onto 0.22-μm Durapore Membrane filters (Millipore), after passing an 8-μm pre-filter, using *in-situ* pumps (WTS-LV, McLane). Upon recovery, filters were immediately frozen and stored at -80°C until further analysis. DNA was extracted using a chloroform-phenol extraction protocol [35]. Sequencing of 2.5-μg DNA samples was done on a GS-FLX 454 pyrosequencer (Roche). A detailed description of the raw read processing as well as the functional (cytochrome oxidase type) and taxonomic sequence assignment is given in the S1 File.

Metagenomic and metatranscriptomic data from the Chilean OMZ (<1.6 μm fraction; Table 1) were provided by F. Stewart and E. DeLong. For further details on sample collection, sequencing and sequence post-processing please refer to reference [34]. Here, BLAST hits for all non-replicate, non-rRNA sequences (DNA and cDNA) were analysed for gene abundance and expression of the various types of cytochrome oxidases and their taxonomic assignments.

## Results and Discussion

### Oxygen distributions

**Fig 1:**
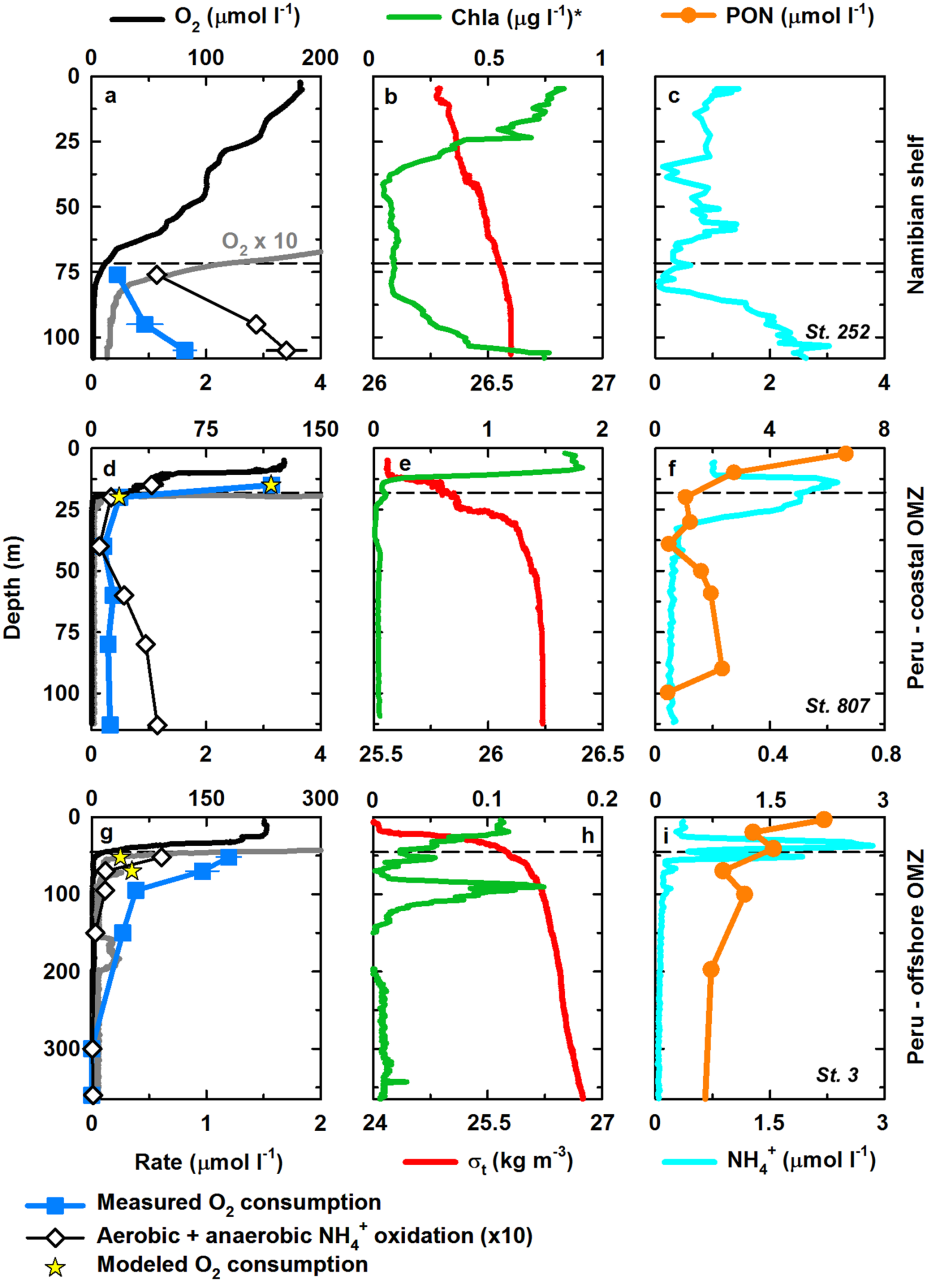
Physicochemical zonation and rates of microbial respiration in the OMZs off Namibia and Peru. (a-c) Namibian shelf (station 252, 111m). (d-f) Peruvian coastal OMZ (station 807, 115 m). (g-i) Offshore Peruvian OMZ (station 3, 4697 m). Dashed lines indicate the upper OMZ boundary (O_2_ ≤15 μmol l^-1^). Previously determined rates of aerobic and anaerobic NH_4_^+^ oxidation [14,24,25] are tenfold magnified. Please note the differences in scale between stations. *Chlorophyll *a* concentrations in panel b in relative units.

The stations investigated on the Namibian shelf (19°S-23°S) were characterized by high surface chlorophyll *a* concentrations, i.e. high primary productivity, at the time of sampling [25]. Oxygen concentrations in the surface waters ranged from ~150 to 250 μmol l^-1^ and gradually declined to ≤15 μmol l^-1^ (here used as a cut-off for the upper OMZ boundary) at ~65–85 m depth (Fig 1a and S1 Fig). At two sampling sites (station 225 and 252), steep O_2_ gradients in the upper OMZ as well as non-detectable levels of O_2_ (≤100 nmol l^-1^) by STOX sensor measurements in the lower OMZ (S1 Table), indicated apparently anoxic conditions over the shelf. In contrast, O_2_ concentrations in the lower micromolar range (~2–6 μmol l^-1^) persisted throughout the OMZ at nearby stations (231 and 243). Both O_2_ and density gradients indicated a rather weak stratification of the Namibian shelf waters, facilitating vertical mixing of more oxygenated surface waters into the OMZ.

In the OMZ off Peru (6°S-16°S), stations covered a wider range of productivity regimes [14]. Sampling sites ranged from highly productive, shallow coastal stations to more oligotrophic, deep offshore ones, as indicated by more than tenfold different chlorophyll *a* levels between stations (Fig 1e and 1h, S1 Fig). Off the central Peruvian coast, surface water O_2_ concentrations were as low as ~125 μmol l^-1^ (station 807) and rapidly declined to ≤15 μmol l^-1^ at ~20–30 m depth. Further offshore, the oxic mixed layer was more extensive, with the upper OMZ boundary typically located at ~50–70 m depth. Non-detectable O_2_ levels in the lower OMZ by STOX sensor measurements (≤50 nmol l^-1^; S1 Table) and cross-calibration of the data obtained by conventional O_2_ sensors with the STOX sensor data, indicated the onset of anoxia ~20–50 m below the OMZ boundary. In agreement, extensive STOX measurements during a recent hydrochemical survey of the South Pacific OMZ showed these waters to be O_2_-depleted down to at least 10 nmol l^-1^ [23]. Generally pronounced pycnoclines indicated a stronger vertical stratification off Peru, as compared to the Namibian shelf OMZ, and thus reduced mixing across the upper OMZ boundary. Further offshore, episodic lateral advection of O_2_-enriched waters into the lower OMZ was however clearly evident from local O_2_ maxima (5–25 μmol l^-1^) at 50–100 m below the oxycline (station 3 and 36; Fig 1g and S1 Fig).

### Oxygen consumption rates

On the Namibian shelf, aerobic respiration was measured at four sampling sites in ^18-18^O_2_ labelling experiments, from the upper OMZ boundary down to the sediment-water interface (S1 Table). Oxygen consumption rates were fairly consistent between stations and depths, typically ranging from ~0.15 to 0.5 μmol O_2_ l^-1^ d^-1^, and showed no significant correlation with *in-situ* O_2_ concentrations (Spearman p >0.05). The latter may, at least in part, owe to the uniform ^18-18^O_2_ adjustments (~2.5 μmol l^-1^) of the incubations irrespective of *in-situ* O_2_ levels, thus stimulating or inhibiting aerobic respiration compared to *in-situ* activity. Noticeably higher rates (~1–1.6 μmol O_2_ l^-1^ d^-1^) were observed at one station (252) in the lower OMZ and the shelf bottom waters. In this zone, enhanced potential for aerobic respiration likely resulted from a high availability of fresh organic matter, as indicated by high chlorophyll *a* levels in the lower OMZ (Fig 1b).

Off Peru, ^18-18^O_2_ labelling experiments were carried out at seven stations, with ^18-18^O_2_ adjustments aimed at mimicking the O_2_ gradient from the upper OMZ boundary towards the OMZ core. Here, the incubations revealed a significant correlation between O_2_ consumption rates and *in-situ* O_2_ concentrations (Spearman R = 0.76, p ≤0.001), i.e. high aerobic respiration at the upper OMZ boundary and rapidly decreasing rates towards the OMZ core (Fig 1d and 1g, S1 Fig). Maximum O_2_ consumption rates in the upper OMZ waters were >3 μmol O_2_ l^-1^ d^-1^ near the Peruvian coast and declined to ~1 μmol O_2_ l^-1^ d^-1^ at the open ocean stations (S1 Table), consistent with observed shelf-offshore gradients in export production for the region [14]. In the lower (anoxic) Peruvian OMZ, potential rates of aerobic respiration showed little variability within and between stations, and typically ranged from ~0.2 to 0.4 μmol O_2_ l^-1^ d^-1^. At the stations furthest offshore, i.e. the least productive ones (station 3 and 5), O_2_ consumption in the core of the OMZ was below the detection limit of the ^18-18^O_2_ labelling approach employed here (~0.1 μmol O_2_ l^-1^ d^-1^).

Given the methodological challenges of accurately determining O_2_ concentrations in the lower micromolar range [29,31], very few direct measurements of aerobic respiration in OMZs exist for comparison. Oxygen consumption rates of similar magnitude (~0.5–2 μmol O_2_ l^-1^ d^-1^), have recently been measured using STOX sensors in low-O_2_ waters of the productive North and South Pacific coastal OMZs [26,31]. Earlier estimates of aerobic respiration in the Eastern Pacific upwelling regions based on particle flux attenuations [36] also compare well with upper OMZ respiration rates determined in this study. Calculating O_2_ flux gradients provides another indirect approach to estimate O_2_ consumption rates. Here, respiration rates were modelled for incubation depths near the upper OMZ boundary at selected stations off Peru (Fig 1d and 1g, S1 Fig). Modelled rates matched measured ones (±5%) at two sites (station 807 and station 36 at 90 m), but were significantly lower (~65–90%) for the remaining sampling locations. Higher experimentally determined O_2_ consumption rates may partially owe to a stimulation of aerobic respiration by slightly elevated ^18-18^O_2_ levels in the incubations compared to O_2_ concentrations *in-situ*. On the other hand, lower modelled respiration rates are expected since the diffusion-controlled 1-D model implies steady state conditions, and balances the rates with the vertical diffusive transport only. Any additional advective O_2_ transport and related transient effects are neglected, resulting in either similar or lower modelled respiration rates compared to measured ones. Indeed, local sub-oxycline O_2_ maxima can be observed at several stations (Fig 1g and S1 Fig), the majority of which are likely associated with advective O_2_ transport. In some instances, locally elevated O_2_ concentrations might indicate photosynthetic O_2_ production within the OMZ (Fig 1g and 1h) [26].

Overall, the lateral distribution of aerobic respiration at the upper OMZ boundaries, i.e. at micromolar O_2_ levels, appears to be largely controlled by the availability of organic matter, as indicated by maximum O_2_ consumption rates in organic-rich coastal waters (Fig 1f and 1i) [14,29]. Less variable, but significant potential for aerobic respiration was also consistently measurable in samples from apparently anoxic depths, particularly in the core of the Peruvian OMZ. Here, actual rates of microbial O_2_ consumption can be expected to primarily depend on the presence or absence of trace O_2_ concentrations. A recent study concluded that the South Pacific OMZ core is largely functionally anoxic and possibly only contains picomolar concentrations of O_2_, due to efficient O_2_ scavenging by microorganisms [23]. At the same time, however, the traditional view of largely static O_2_-deficient zones increasingly shifts towards temporally more dynamic OMZs. Large-scale observations indicate episodic O_2_ injections into the Peruvian OMZ via intrusions of oxygenated surface waters or mixing events, such as related to eddy activity [27,28]. Further, O_2_-bearing water layers surrounded by hundreds of meters of anoxic waters indicate lateral O_2_ supply into the OMZs (Fig 1g) [26]. These pulses of O_2_ can, at least temporarily, sustain aerobic respiration in otherwise anoxic waters. The total flux of O_2_ via episodic O_2_ injections is difficult to assess since the frequency of observations of sub-oxycline O_2_ maxima is likely reduced due to dispersion and efficient microbial O_2_ consumption. However, the importance of such transient O_2_ transport is indicated by recent biogeochemical modelling of the South Pacific OMZ, which hints at substantial aerobic organic matter degradation in the OMZ, and lateral advection as an important source of O_2_ for aerobic respiration therein [37].

### Aerobic terminal oxidases gene abundance and expression

**Fig 2:**
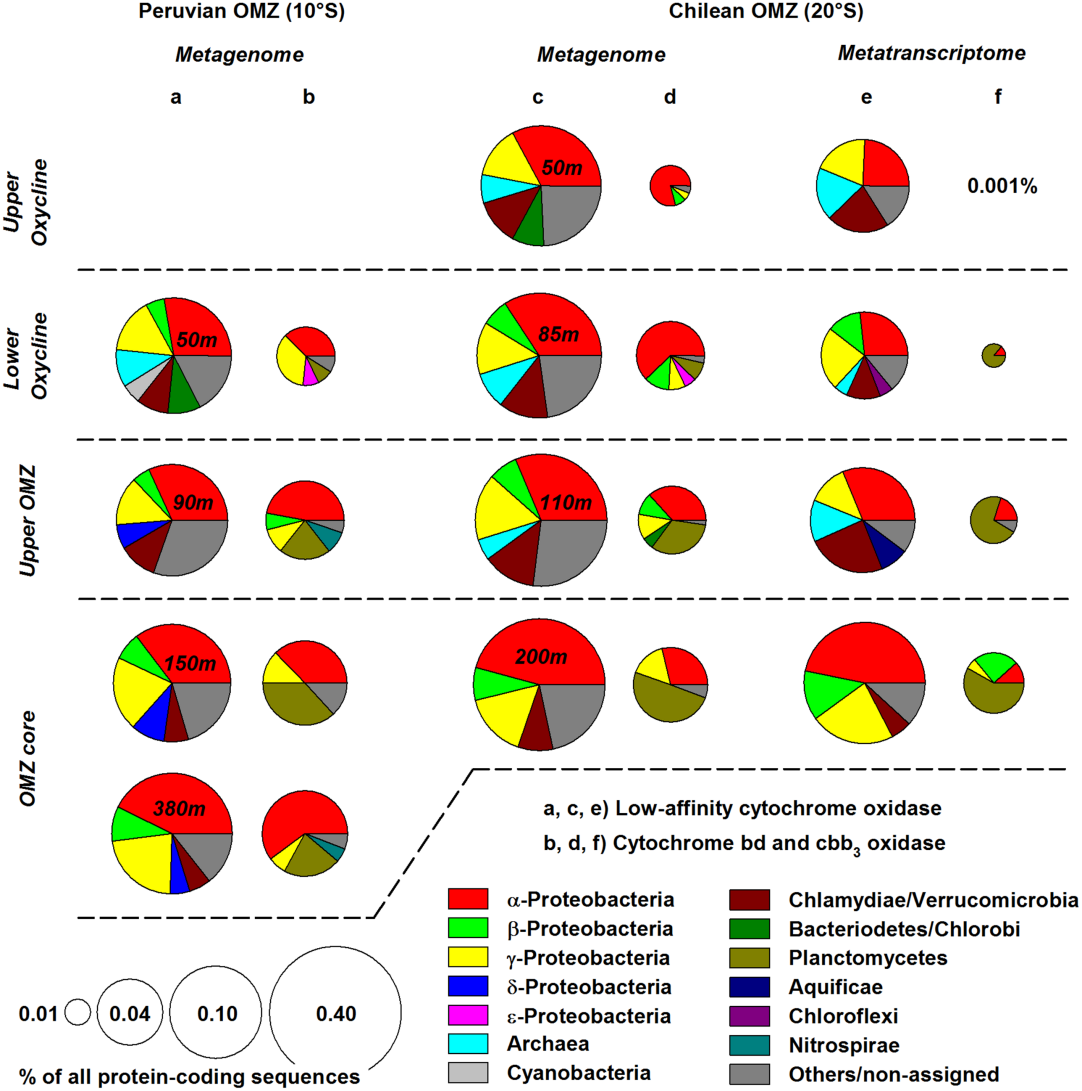
Abundance of genes and transcripts encoding for terminal respiratory oxidases in the ETSP OMZ. (a, b) Abundance of low-affinity (cytochrome c oxidase) and high-affinity (cytochrome bd and cbb_3_ oxidase) aerobic oxidases in the Peruvian OMZ (station 3). (c-f) Abundance and expression of cytochrome oxidase genes in the OMZ off Chile during cruise MOOMZ-1 [34]. Taxonomic affiliations of cytochrome oxidases are shown on domain, phylum or class level if represented by at least 5% of oxidase-coding sequences. Exact abundance and expression levels as well as taxonomic assignments of the individual types of cytochrome oxidases are given in S3 Table.

Identification of cytochrome oxidases, catalysing the terminal electron transfer during oxic respiration, in the South Pacific OMZ provided further evidence for active aerobic respiration at near-anoxic levels of O_2_. Metagenomes obtained in the Peruvian offshore OMZ (station 3) as well as metagenomes and metatranscriptomes previously collected off Chile [34], revealed presence and expression of genes encoding for low-affinity cytochrome *c* oxidases (K_m_ ~200 nmol O_2_ l^-1^) as well as high-affinity cytochrome *bd* and *cbb*_*3*_ oxidases (K_m_ <10 nmol O_2_ l^-1^) [38] from the oxycline down to the OMZ core (Fig 2 and S3 Table).

In all samples investigated, the majority of aerobic oxidase genes and gene transcripts were of the common low-affinity type (68–99%). Cytochrome *c* oxidases could largely be assigned to the phylum of Proteobacteria (mostly Alpha-, Beta- and Gammaproteobacteria), which is generally dominant in OMZs [39], followed by the Chlamydiae-Verrucomicrobia group. Consistent with a prior study [34], one of the most abundant taxa was the heterotrophic marine genus *Pelagibacter* (Alphaproteobacteria), accounting for up to 12% and 15% of all respiratory oxidase genes and gene transcripts, respectively, in the Chilean OMZ oxycline. In general, heterotrophs appeared to dominate the low-affinity type microbial community, yet also a number of cytochrome *c* oxidases strongly similar to those of marine chemo- and photoautotrophic prokaryotes were identified. Most notably, cytochrome *c* oxidases of the archaeal NH_4_^+^ oxidizer *Nitrosopumilus maritimus* were highly abundant and expressed (2–5% and 4–15% of all aerobic oxidase DNA and RNA sequences, respectively) in the oxycline and upper OMZ off Peru and Chile. In this zone, *N*. *maritimus* abundances, NH_4_^+^-oxidizing activity as well as archaeal ammonia monooxygenase subunit A gene and transcript numbers are generally elevated [14,17,34,40,41]. Further, low-affinity oxidase genes and gene transcripts of NH_4_^+^-oxidizing (*Nitrosococcus* and *Nitrosomonas*) and NO_2_^-^-oxidizing bacteria (*Nitrobacter*, *Nitrococcus and Nitrospira*) were detected throughout the OMZ, in line with active bacterial nitrification in the South Pacific [14,17,42] and other oceanic OMZs [25,43,44]. In addition, DNA and RNA sequences collected in the Peruvian and Chilean OMZ, respectively, matched cyanobacterial cytochrome *c* oxidases, mostly of the genus *Prochlorococcus*; off Peru, noticeably high abundances (1–5% of all aerobic oxidase DNA sequences) coincided with the deep chlorophyll *a* maximum (Fig 1h) as well as previously identified *Prochlorococcus*-specific marker pigments [45] at this station. These low-light-adapted cyanobacteria are widely distributed across the major OMZs [46,47], and likely also provide a local source of O_2_ via oxygenic photosynthesis well below the oxycline [26].

A large variety of aerobic and anaerobic microorganisms have adapted to microoxic environments by evolving terminal respiratory oxidases with high O_2_ affinities [38]. For example, the cytochrome *bd* oxidase of *Escherichia coli* with an apparent K_m_ value of 3–8 nmol O_2_ l^-1^ is maximally expressed under microaerobic conditions [48,49], permitting aerobic growth at lower nanomolar, possibly even picomolar O_2_ concentrations [50]. Another high-affinity respiratory oxidase, the cytochrome *cbb*_*3*_ oxidase, has a similarly low K_m_ value of 7 nmol O_2_ l^-1^ [51]. Off the Peruvian and Chilean coasts, DNA and RNA sequences of both types of high-affinity oxidases could mostly be assigned to auto- and heterotrophic alpha-, beta and gammaproteobacterial taxa (e.g. *Rosebacter*, *Ralstonia* and sulphur-oxidizing symbionts, respectively) as well as Planctomycetes (Fig 2 and S3 Table). In the OMZ and lower oxycline, cytochrome *bd* and *cbb*_*3*_ oxidases closely resembling those of the anammox bacterium Candidatus *Kuenenia stuttgartiensis* (Planctomycetes) were present in particularly high abundances (up to 49% and 86% of all high-affinity type DNA and RNA sequences, respectively). Presumably, the O_2_-sensitive anammox bacteria [52] use high-affinity cytochrome oxidases as a means of detoxification [53], enabling them to remain active in a broader O_2_ regime [24]. Further, in the Peruvian OMZ metagenomes on average 6% of high-affinity oxidases identified were strongly similar to the cytochrome *bd* oxidase of the NO_2_^-^ oxidizer Candidatus *Nitrospira defluvii*, in line with active NO_2_^-^ oxidation at sub-micromolar O_2_ levels in the South Pacific OMZ [14,23].

Along the O_2_ gradients investigated, the relative abundance of aerobic oxidase genes and gene transcripts increased from the oxycline towards the lower OMZ. High-affinity cytochrome oxidases were particularly enriched in (near) anoxic zone samples, on both DNA and RNA level. Off Peru and Chile, the ratio of high-affinity to low-affinity oxidase-coding genes increased from 0.14 (50 m) to 0.31 (90–380 m) and 0.01 (50 m) to 0.12 (85-200m), respectively. Most notably, cytochrome *bd* and *cbb*_*3*_ oxidase transcripts in the Chilean OMZ accounted for only 0.001% of all protein-coding sequences at the upper oxycline (~100 μmol O_2_ l^-1^) [34], while showing a 30-fold higher relative abundance (0.03%) in the presumably anoxic OMZ core (Fig 2f). A recent metatranscriptomic study of a sulphidic event off central Peru, similarly revealed a distinct trend of increasing cytochrome *cbb*_*3*_ oxidase expression as O_2_ concentrations decreased [54]. Intriguingly, the ratio of high-affinity to low-affinity oxidase transcripts peaked at the lower oxycline and upper OMZ (0.17; 0 < O_2_ ≤10 μmol l^-1^). At the more oxygenated upper oxycline and at the OMZ core, where the diffusive O_2_ flux can be expected to equal zero, high-affinity type fractions were significantly reduced (0.12 and 0.04, respectively). Enhanced expression of high-affinity oxidases in the microoxic transition zone overlying the O_2_-depleted OMZ core clearly demonstrates the capacity of microorganisms to exploit sub-micromolar O_2_ concentrations. In corroboration, apparent K_m_ values for aerobic respiration of 10–200 nmol O_2_ l^-1^ have recently been reported for the North and South Pacific OMZs [26,31]. Further, identification of cytochrome oxidase genes and gene transcripts revealed broadly similar microbial community compositions between the Chilean and Peruvian OMZ, suggesting microaerobic respiration to be a universal feature of oceanic OMZs. Hence, although O_2_ concentrations in OMZs remain largely below detection, O_2_-dependent nitrification and heterotrophic aerobic respiration still proceed, due to efficient O_2_ scavenging of microbial communities that are well adapted to these (transiently) microoxic environments.

### Oxygen sensitivity of aerobic respiration

**Fig 3:**
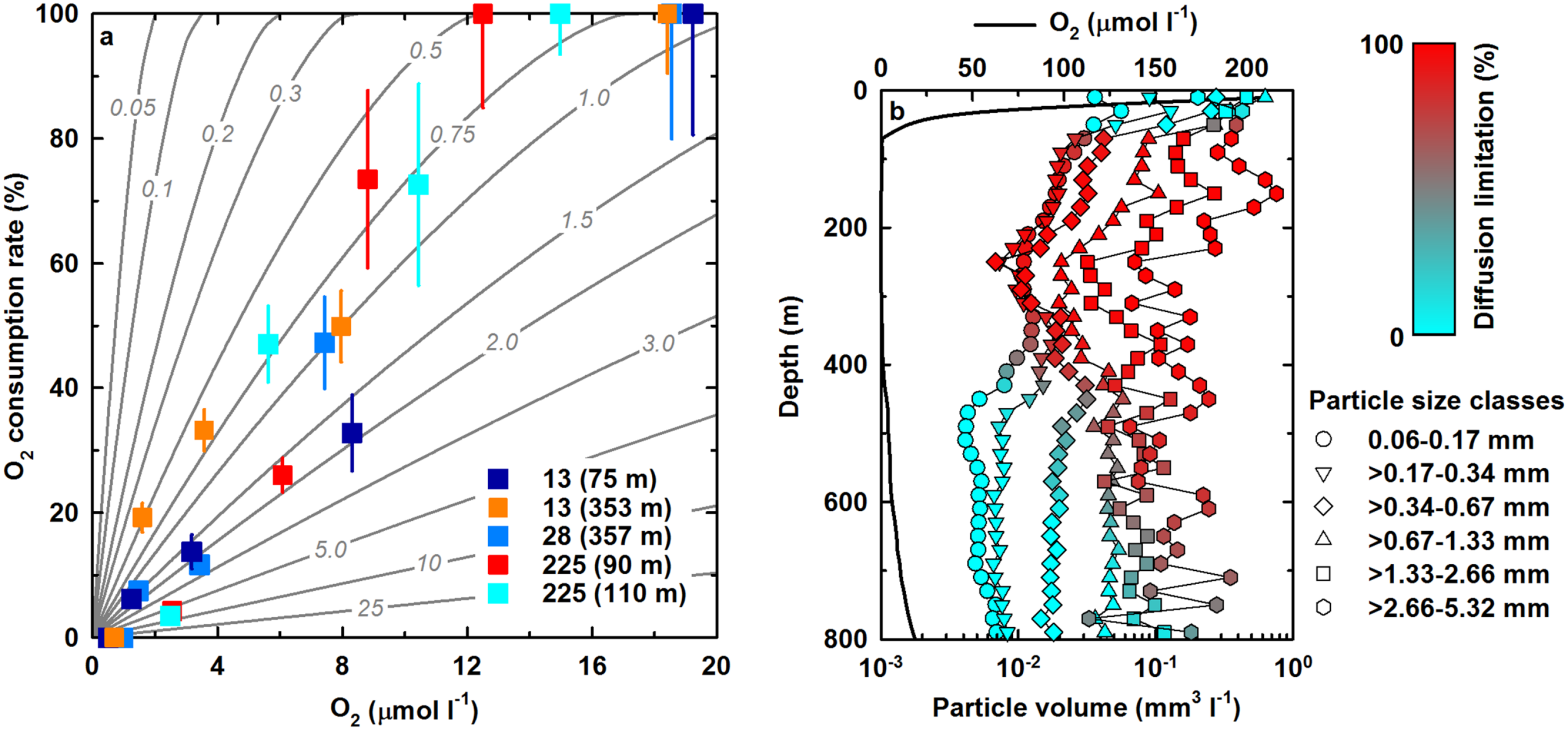
Oxygen sensitivity of aerobic respiration and OMZ particle size distributions. (a) O_2_ sensitivity assays in the Namibian (station 225) and Peruvian OMZ (stations 13 and 28) during cruises M76 and M77-3, respectively. Oxygen consumption rates are given as percentages of the highest rate observed (= 100%) among all O_2_ treatments (see S2 Table for absolute rates). Error bars for O_2_ consumption rates are standard errors calculated from linear regression. Isolines (grey) indicate diffusion-limited respiration rates inside aggregates of 0.01–25 mm in diameter. A detailed description of how aggregate-size-dependent rates were calculated is included in the S1 File. (b) Vertical distribution of particle volumes (20 m bins) for six size classes between 0.06 and 5.32 mm (ESD) in the central Peruvian OMZ (12.62°S/77.55°W) during cruise M93. Color shading indicates diffusion limitation of aerobic respiration inside particles. For clarity, particles >5.32 mm are not depicted here. A more general overview of particle size distributions in the ETSP OMZ is given in S2 Fig.

At the upper OMZ boundaries, steep O_2_ gradients mark the transition from oxic to anoxic environments. Experiments simulating changing O_2_ concentrations (~0.5–20 μmol l^-1^) in this transition zone, revealed a near-linear decrease of O_2_ consumptions rates with decreasing levels of O_2_ in the Namibian and Peruvian OMZ (Fig 3a and S2 Table); a surprising result, given that K_m_ values of aerobic respiratory oxidases are two to three orders of magnitude lower than ambient O_2_ concentrations in the incubations. Recent studies targeting aerobic NH_3_ and NO_2_^-^ oxidation, showed both processes to be also mostly insensitive to decreasing O_2_ concentrations over the same range [14,24,25]. Assuming O_2_ consumption in our experiments to be largely coupled to heterotrophic activity, the apparent high O_2_ sensitivity observed here may result from O_2_ diffusion limitation of aggregate-associated organic matter respiration [29].

High fluxes of particulate matter [36] and pronounced O_2_ deficiency in OMZs provide ideal conditions for the development of O_2_-depleted microenvironments [55,56]. Anoxic microniches inside marine snow aggregates have been suggested to exist at environmental O_2_ concentrations up to several tens of micromoles [57–59], thereby extending the effective anoxic OMZ volume [24]. *In-situ* particle size spectra in the Peruvian OMZ show a large fraction of particles larger than ~0.9 mm (Fig 3b, S2 Fig), above which size-dependent model results indicate diffusion-limited respiration for O_2_ ≤20 μmol l^-1^ (Fig 3a and 3b). Moreover, organic-rich aggregates are hot-spots of microbial activity [60], and large fractions of heterotrophic taxa in OMZs are particle-associated [61,62]. Hence, actual O_2_ levels encountered by much of the aerobic heterotrophic microbial community may be significantly lower than measured bulk concentrations. Likewise, anaerobic microorganisms that can be found in association with particles in OMZs, such as anammox bacteria [61], may in fact be exposed to O_2_ concentrations much lower than ambient levels. Indeed, a recent study revealed a tight coupling of aerobic and anaerobic N-cycling processes within cyanobacterial aggregates, and suggests aggregates to be important sites of N-loss at low ambient O_2_ [59]. Oxygen-reduced microniches might explain the observed apparent low O_2_ affinity and high O_2_ tolerance (>>1 μmol l^-1^) of aerobic and anaerobic microorganisms in OMZs, respectively, under stagnant experimental conditions [24]. Their true K_m_ values for O_2_ as well as O_2_ sensitivities are more likely in the nanomolar range, i.e. closer to those reported from culture studies and stirred environmental samples, in which microbial O_2_ exposure equals ambient O_2_ levels [26,38,50,52,63].

### Ammonium release by microaerobic organic matter respiration

Recent reports from major oceanic OMZs have shown that rates of both aerobic and anaerobic NH_4_^+^ oxidation (anammox) often peak near the upper OMZ boundary [13,14,17]. At the same time, anaerobic sources of NH_4_^+^ are generally insufficient to fully explain ammonium oxidation rates in this zone. Particularly denitrification, traditionally regarded as the major N-remineralization pathway in OMZs, remained largely undetectable in these studies. In light of so-far unidentified sources of NH_4_^+^, the overall significance of anammox in OMZ N-loss has been questioned [64]. Instead, spatial variability in organic matter fluxes as well as in organic matter stoichiometry have been suggested to result in patchy and thus often missed denitrifying activity [16,65]. We compared the O_2_ consumption rates determined in this study with previously reported N-cycling rates for the same sampling sites [14,24,25]. In contrast to the proposed large-scale constraint of anammox activity by N-release via denitrification [16], our data suggest ammonium oxidation in the upper OMZs to be largely coupled to microaerobic organic matter remineralization.

Enhanced O_2_ consumption in the upper Peruvian OMZ coincided with a marked decrease in PON and concomitantly high NH_4_^+^ concentrations (~0.5–2 μmol l^-1^) in this zone (Fig 1f and 1i, S1 Fig), clearly indicating organic matter remineralization via aerobic respiration. For both the upper Peruvian and Namibian OMZ, on average 80% of measured O_2_ consumption were estimated to be due to the activity of heterotrophic microorganisms, when taking into account O_2_ consumption via aerobic NH_4_^+^ and NO_2_^-^ oxidation (Table 2). Compared to anaerobic remineralization pathways in this zone, these heterotrophic O_2_ consumption rates accounted for ~45–100% of organic matter degradation (assuming Redfield stoichiometry: C/N = 6.6), with the remainder mainly attributable to NO_3_^-^ reduction to NO_2_^-^. Further, in waters off both Namibia and Peru rates of heterotrophic oxic respiration, and thus aerobic NH_4_^+^ release, were significantly correlated to rates of total (aerobic + anaerobic) NH_4_^+^ oxidation (Spearman R = 0.69 and 0.50, respectively, p <0.01), emphasizing the tight coupling of NH_4_^+^ producing and consuming processes in OMZs (Fig 1a, 1d and 1g, S1 Fig). Near the upper OMZ boundaries, aerobic as well as anaerobic NH_4_^+^ sources and sinks resulted in net rates of -36 to 365 nmol NH_4_^+^ l^-1^ d^-1^ (Table 2). Aerobic organic matter respiration could on average account for 91% of the NH_4_^+^ production.

**Table 2:** Ammonium budget for the upper Namibian and Peruvian OMZ considering aerobic and anaerobic NH_4_^+^-producing and consuming processes. For the sake of clarity, standard errors for the individual processes determined at each station are not listed here (typically ~10% of the measured rate). Directly measured rates are in italics, the remainder were inferred from idealized stoichiometries (see S1 File for further details). Liberation of NH_4_^+^ from organic matter via oxic as well as NO_3_^-^ respiration accounts for bacterial N-uptake assuming a growth efficiency of 0.15 [66] and a C/N ratio of 6.6 for the heterotrophic community [67,68].

	Namibian OMZ		Peruvian OMZ						
**Station**	243	252	805	807	811	3^4^	5	13	36
**Site characteristics**									
Water depth (m)	103	111	999	115	145	4,697	4,525	356	2,845
Depth sampled (m)	80	76	62	15	54	52	75	38	90
O_2_ (μmol l^-1^)	7.59	1.11	7.46	~20	4.16	4.01	2.60	3.40	1.49
NH_4_^+^ (μmol l^-1^)	0.00	0.12	0.27	0.58	0.05	1.27	0.07	0.10	0.05
**Aerobic N cycling (nmol N l** ^**-1**^ **d** ^**-1**^ **)**									
*NH*_*3*_ *oxidation* ^1^	21	93	89	49	13	60	5.8	14	35
*NO*_*2*_^*-*^ *oxidation* ^1^	74	112	38	928	70	35	32	29	186
**O**_**2**_ **consumption (nmol O**_**2**_ **l** ^**-1**^ **d** ^**-1**^ **)**									
*Total oxic respiration*	230	450	541	3,136	605	1,195	730	990	1,060
Heterotrophic oxic respiration^2^	161	254	389	2,599	552	1087	706	954	915
**Anaerobic N cycling (nmol N l** ^**-1**^ **d** ^**-1**^ **)**									
*NO*_*3*_^*-*^ *reduction* ^1^	17	370	18	1,010	0.0	40	0.0	0.0	42
*DNRA* ^1^	0.0	12	0.3	1.1	0.0	0.0	0.0	0.0	0.8
*Anammox* ^1^	25	42	0.0	112	9.6	1.6	4.0	3.6	2.3
**NH**_**4**_^**+**^ **sinks (nmol NH**_**4**_^**+**^ **l** ^**-1**^ **d** ^**-1**^ **)**									
*NH*_*3*_ *oxidation*	-21	-93	-89	-49	-13	-60	-5.8	-14	-35
*Anammox*	-13	-21	0.0	-56	-4.8	-0.8	-2.0	-1.8	-1.2
**NH**_**4**_^**+**^ **sources (nmol NH**_**4**_^**+**^ **l** ^**-1**^ **d** ^**-1**^ **)**									
Heterotrophic oxic respiration^3^	21	33	50	333	71	139	91	122	117
NO_3_^-^ reduction^3^	1.1	24	1.1	65	0	2.5	0	0	2.7
DNRA^3^	0	17	0.5	1.6	0	0	0	0	0.9
**Net NH**_**4**_^**+**^ **rate (nmol l** ^**-1**^ **d** ^**-1**^ **)**	-12 (±11)	-45 (±30)	-37 (±20)	295 (±76)	53 (±22)	81 (±20)	83 (±11)	107 (±22)	85 (±22)

^1^ From references [14,24,25].

^2^ Heterotrophic oxic respiration = Total oxic respiration– 1.5 * NH_3_ oxidation– 0.5 * NO_2_^-^ oxidation.

^3^ Heterotrophic oxic respiration: O_2_/NH_4_^+^ = 106/16; NO_3_^-^ reduction: NO_3_^-^/NH_4_^+^ = 212/16; DNRA: NO_3_^-^/NH_4_^+^ = 53/69.

^4^ Station sampled for metagenomic analysis (Fig 2).

Obviously, estimates of NH_4_^+^ liberation from organic matter are highly sensitive to changes in the C/N ratio. Considering non-Redfieldian C/N ratios of 5.3 and 10.6, as observed for surface particulate matter in the Peruvian OMZ [45], results in an increase of 55% and a decrease of 61%, respectively, in aerobic NH_4_^+^ release (assuming a constant O_2_/C ratio). Regardless of the C/N scenario chosen, sufficient NH_4_^+^ is provided to fuel 90–100% of the combined demands of aerobic and anaerobic NH_4_^+^ oxidation at five out of six stations in the Peruvian OMZ. More negative NH_4_^+^ balances are observed for the shallow Namibian shelf OMZ. Here, sedimentary NH_4_^+^ release likely plays a more important role in driving N-loss via anammox (Fig 1c).

## Conclusions

In summary, extensive rate measurements combined with metagenomic as well as metatranscriptomic analyses show widespread potential for microaerobic respiration in the Namibian and South Pacific OMZs. Microorganisms inhabiting the OMZ use high-affinity respiratory oxidases to exploit traces amounts of O_2_, brought in via intrusions of oxygenated surface waters, lateral advection of O_2_-bearing water masses, or produced locally by low-light adapted phytoplankton. At the upper OMZ boundary, where micromolar O_2_ concentrations persist, microaerobic respiration is the major mode of organic matter degradation and primary source of NH_4_^+^ for aerobic NH_4_^+^ oxidation and N-loss via anammox. The close spatial coupling of aerobic and anaerobic pathways in (O_2_-carrying) OMZ waters is likely facilitated by formation of O_2_-reduced microniches in sinking aggregates.

In current biogeochemical models, remineralization of organic matter exported to the OMZs is largely coupled to denitrification, typically resulting in overestimated N-loss from tropical and subtropical upwelling systems [22,69]. Denitrification by facultative anaerobic heterotrophs, however, only occurs under (near) anoxic conditions [63], and a large fraction of sinking organic matter is remineralized in more oxic upper OMZ waters. Considering aerobic microbial respiration as a major mode of remineralization of export production in this zone might help to improve model-based assessments of the current oceanic N-balance [37], as well as the effects of globally expanding OMZs and changing productivities on the ocean’s future N-budget.

## Supporting Information

S1 File (PDF)

S1 Fig Physicochemical zonation and rates of microbial respiration in the OMZs off Namibia and Peru. (PDF)

S2 Fig Particle size distributions in the South Pacific OMZ. (PDF)

S1 Table Oxygen consumption rates in the OMZs off Namibia and Peru. (PDF)

S2 Table O_2_ sensitivity assays in the OMZs off Namibia and Peru. (PDF)

S3 Table Abundance and taxonomic assignment of BLAST hits for low-affinity and high-affinity (bd and cbb_3_) cytochrome oxidases of metagenomes and metatranscriptomes from the South Pacific OMZ. (PDF)

## References

[1] Lam P, Kuypers MMM. Microbial Nitrogen Cycling Processes in Oxygen Minimum Zones. Ann Rev Mar Sci. 2011;3: 317–347. 21329208 10.1146/annurev-marine-120709-142814

[2] Helly JJ, Levin LA. Global distribution of naturally occurring marine hypoxia on continental margins. Deep Res. 2004;51: 1159–1168.

[3] Karstensen J, Stramma L, Visbeck M. Oxygen minimum zones in the eastern tropical Atlantic and Pacific oceans. Progr Ocean. 2008;77: 331–350.

[4] Gruber N. The Dynamics of the Marine Nitrogen Cycle and its Influence on Atmospheric CO2 In: Follows M, Oguz T, editors. The ocean carbon cycle and climate, NATO ASI Series. Dordrecht: Kluwer Academic; 2004 pp. 97–148.

[5] Moore CM, Mills MM, Arrigo KR, Berman-Frank I, Bopp L, Boyd PW, et al Processes and patterns of oceanic nutrient limitation. Nat Geosci. 2013;6: 701–710.

[6] Cline JD, Richards FA. Oxygen Deficient Conditions and Nitrate Reduction in the Eastern Tropical North Pacific Ocean. Limnol Ocean. American Society of Limnology and Oceanography; 1972;17: 885–900.

[7] Codispoti LA, Packard TT. Denitrification Rates in the Eastern Tropical South-Pacific. J Mar Res. 1980;38: 453–477.

[8] Naqvi SWA. Some aspects of the oxygen-deficient conditions and denitrification in the Arabian Sea. J Mar Res. 1987;45: 1049–1072.

[9] Zumft WG. Cell Biology and Molecular Basis of Denitrification. Microbiol Molec Biol Rev. 1997;61: 533–616. 9409151 10.1128/mmbr.61.4.533-616.1997 PMC232623

[10] Kuypers MMM, Lavik G, Woebken D, Schmid M, Fuchs BM, Amann R, et al Massive nitrogen loss from the Benguela upwelling system through anaerobic ammonium oxidation. PNAS. 2005;102: 6478–6483. 15843458 10.1073/pnas.0502088102 PMC556276

[11] Thamdrup B, Dalsgaard T, Jensen MM, Ulloa O, Farias L, Escribano R. Anaerobic ammonium oxidation in the oxygen-deficient waters off northern Chile. Limnol Ocean. 2006;51: 2145–2156.

[12] Hamersley MR, Lavik G, Woebken D, Rattray JE, Lam P, Hopmans EC, et al Anaerobic ammonium oxidation in the Peruvian oxygen minimum zone. Limnol Ocean. 2007;52: 923–933.

[13] Jensen MM, Lam P, Revsbech NP, Nagel B, Gaye B, Jetten MSM, et al Intensive nitrogen loss over the Omani Shelf due to anammox coupled with dissimilatory nitrite reduction to ammonium. ISME J. International Society for Microbial Ecology; 2011;5: 1660–1670. 10.1038/ismej.2011.44 PMC3176517 21509044

[14] Kalvelage T, Lavik G, Lam P, Contreras S, Arteaga L, Löscher CR, et al Nitrogen cycling driven by organic matter export in the South Pacific oxygen minimum zone. Nat Geosci. 2013;6: 228–234.

[15] Dalsgaard T, Canfield DE, Petersen J, Thamdrup B, Acuna-Gonzalez J. N2 production by the anammox reaction in the anoxic water column of Golfo Dulce, Costa Rica. Nature. 2003;422: 606–608. 12686998 10.1038/nature01526

[16] Babbin AR, Keil RG, Devol AH, Ward BB. Organic Matter Stoichiometry, Flux, and Oxygen Control Nitrogen Loss in the Ocean. Science. 2014;344: 406–408. 10.1126/science.1248364 24763588

[17] Lam P, Lavik G, Jensen MM, van De Vossenberg J, Schmid M, Woebken D, et al Revising the nitrogen cycle in the Peruvian oxygen minimum zone. PNAS. 2009;106: 4752–4757. 10.1073/pnas.0812444106 19255441 PMC2649953

[18] Lipschultz F, Wofsy SC, Ward BB, Codispoti LA, Friedrich G, Elkins JW. Bacterial transformations of inorganic nitrogen in the oxygen-deficient waters of the Eastern Tropical South Pacific Ocean. Deep Res. 1990;37: 1513–1541.

[19] Canfield DE, Stewart FJ, Thamdrup B, De Brabandere L, Dalsgaard T, Delong EF, et al A Cryptic Sulfur Cycle in Oxygen-Minimum Zone Waters off the Chilean Coast. Science. 2010;330: 1375–1378. 10.1126/science.1196889 21071631

[20] Dalsgaard T, Thamdrup B, Farıas L, Revsbech NP. Anammox and denitrification in the oxygen minimum zone of the eastern South Pacific. Limnol Ocean. 2012;57: 1331–1346.

[21] De Brabandere L, Canfield DE, Dalsgaard T, Friederich GE, Revsbech NP, Ulloa O, et al Vertical partitioning of nitrogen-loss processes across the oxic-anoxic interface of an oceanic oxygen minimum zone. Environ Microbiol. 2013; 1462–2920. 10.1111/1462-2920.12255 24118779

[22] Paulmier A, Kriest I, Oschlies A. Stoichiometries of remineralisation and denitrification in global biogeochemical ocean models. Biogeosciences. 2009;6: 2539–2566.

[23] Thamdrup B, Dalsgaard T, Revsbech NP. Widespread functional anoxia in the oxygen minimum zone of the eastern South Pacific. Deep Res I. Elsevier; 2012;65.

[24] Kalvelage T, Jensen MM, Contreras S, Revsbech NP, Lam P, Günter M, et al Oxygen Sensitivity of Anammox and Coupled N-Cycle Processes in Oxygen Minimum Zones. PLoS One. 2011;6: e29299 10.1371/journal.pone.0029299 22216239 PMC3247244

[25] Füssel J, Lam P, Lavik G, Jensen MM, Holtappels M, Günter M, et al Nitrite oxidation in the Namibian oxygen minimum zone. ISME J. 2012;6: 1200–1209. 10.1038/ismej.2011.178 22170426 PMC3358024

[26] Tiano L, Garcia-Robledo E, Dalsgaard T, Devol AH, Ward BB, Ulloa O, et al Oxygen distribution and aerobic respiration in the north and south eastern tropical Pacific oxygen minimum zones. Deep Sea Res I. 2014;94: 173–183.

[27] Whitmire AL, Letelier RM, Villagrán V, Ulloa O. Autonomous observations of in vivo fluorescence and particle backscattering in an oceanic oxygen minimum zone. Opt Express. 2009;17: 21992–22004. 10.1364/OE.17.021992 19997444

[28] Bertrand A, Ballón M, Chaigneau A. Acoustic Observation of Living Organisms Reveals the Upper Limit of the Oxygen Minimum Zone. PLoS One. 2010;5: e10330 10.1371/journal.pone.0010330 20442791 PMC2862015

[29] Holtappels M, Tiano L, Kalvelage T, Lavik G, Revsbech NP, Kuypers MMM. Aquatic Respiration Rate Measurements at Low Oxygen Concentrations. Ivanovic Z, editor. PLoS One. 2014;9: e89369 10.1371/journal.pone.0089369 24586724 PMC3929708

[30] Holtappels M, Kuypers MMM, Schlüter M, Brüchert V. Measurement and interpretation of solute concentration gradients in the benthic boundary layer. Limnol Ocean Methods. 2011;9: 1–13.

[31] Revsbech NP, Larsen LH, Gundersen J, Dalsgaard T, Ulloa O, Thamdrup B. Determination of ultra-low oxygen concentrations in oxygen minimum zones by the STOX sensor. Limnol Ocean Methods. 2009;7: 371–381.

[32] Holmes RM, Aminot A, Keroul R, Hooker BA, Peterson BJ. A simple and precise method for measuring ammonium in marine and freshwater ecosystems. Can J Fish Aquat Sci. 1999;56: 1801–1808.

[33] Picheral M, Guidi L, Stemmann L, Karl DM, Iddaoud G, Gorsky G. The Underwater Vision Profiler 5: An advanced instrument for high spatial resolution studies of particle size spectra and zooplankton. Limnol Ocean Methods. 2010;8: 462–473.

[34] Stewart FJ, Ulloa O, Delong EF. Microbial metatranscriptomics in a permanent marine oxygen minimum zone. Environ Microbiol. 2011;14: 23–40. 10.1111/j.1462-2920.2010.02400.x 21210935

[35] Weiland N, Löscher C, Metzger R, Schmitz R. Construction and Screening of Marine Metagenomic Libraries In: Streit WR, Rolf D, editors. Methods in Molecular Biology. Humana Press; 2010 pp. 51–56. 10.1007/978-1-60761-823-2_3 20830555

[36] Suess E. Particulate Organic-Carbon Flux in the Oceans—Surface Productivity and Oxygen Utilization. Nature. 1980;288: 260–263.

[37] Su B, Pahlow M, Wagner H, Oschlies A. What prevents nitrogen depletion in the oxygen minimum zone of the eastern tropical South Pacific? Biogeosciences. 2015;12: 1113–1130.

[38] Morris RL, Schmidt TM. Shallow breathing: bacterial life at low O2. Nat Rev Microbiol. 2013;11: 205–212. 10.1038/nrmicro2970 23411864 PMC3969821

[39] Wright JJ, Konwar KM, Hallam SJ. Microbial ecology of expanding oxygen minimum zones. Nat Rev Microbiol. 2012;10: 381–394. 10.1038/nrmicro2778 22580367

[40] Belmar L, Molina V, Ulloa O. Abundance and phylogenetic identity of archaeoplankton in the permanent oxygen minimum zone of the eastern tropical South Pacific. FEMS Microbiol Ecol. 2011;78: 314–326. 10.1111/j.1574-6941.2011.01159.x 21696407

[41] Löscher CR, Kock A, Koenneke M, LaRoche J, Bange HW, Schmitz RA. Production of oceanic nitrous oxide by ammonia-oxidizing archaea. Biogeosciences. 2012;9: 2419–2429.

[42] Ward BB, Glover HE, Lipschultz F. Chemoautotrophic Activity and Nitrification in the Oxygen Minimum Zone off Peru. Deep Res. 1989;36: 1031–1051.

[43] Beman JM, Shih JL, Popp BN. Nitrite oxidation in the upper water column and oxygen minimum zone of the eastern tropical North Pacific Ocean. ISME J. 2013;7: 2192–2205. 10.1038/ismej.2013.96 23804152 PMC3806268

[44] Lam P, Jensen MM, Kock A, Lettmann KA, Plancherel Y, Lavik G, et al Origin and fate of the secondary nitrite maximum in the Arabian Sea. Biogeosciences. 2011;8: 1565–1577.

[45] Franz J, Krahmann G, Lavik G, Grasse P, Dittmar T, Riebesell U. Dynamics and stoichiometry of nutrients and phytoplankton in waters influenced by the oxygen minimum zone in the eastern tropical Pacific. Deep Res I. 2012;62: 20–31.

[46] Goericke R, Olson RJ, Shalapyonok A. A novel niche for Prochlorococcus sp. in low-light suboxic environments in the Arabian Sea and the Eastern Tropical North Pacific. Deep Res I. 2000;47: 1183–1205.

[47] Lavin P, González B, Santibáñez JF, Scanlan DJ, Ulloa O. Novel lineages of Prochlorococcus thrive within the oxygen minimum zone of the eastern tropical South Pacific. Environ Microbiol Rep. 2010;2: 728–738. 10.1111/j.1758-2229.2010.00167.x 23766277

[48] D´mello R, Hill S, Poolel RK. The cytochrome bd quinol oxidase in Escherichia coli has an extremely high oxygen affinity and two oxygen-binding haems: implications for regulation of activity in vivo by oxygen inhibition. Microbiology. 1996;142: 755–763. 8936304 10.1099/00221287-142-4-755

[49] Tseng CP, Albrecht J, Gunsalus RP. Effect of microaerophilic cell growth conditions on expression of the aerobic (cyoABCDE and cydAB) and anaerobic (narGHJI, frdABCD, and dmsABC) respiratory pathway genes in Escherichia coli. J Bacteriol. 1996;178: 1094–1098. 8576043 10.1128/jb.178.4.1094-1098.1996 PMC177770

[50] Stolper DA, Revsbech NP, Canfield DE. Aerobic growth at nanomolar oxygen concentrations. PNAS. 2010;107: 18755–18760. 10.1073/pnas.1013435107 20974919 PMC2973883

[51] Preisig O, Zufferey R, Appleby CA, Thöny-Meyer L, Hennecke H. A high-affinity cbb3-type cytochrome oxidase terminates the symbiosis-specific respiratory chain of Bradyrhizobium japonicum. J Bacteriol. 1996;178: 1532–1538. 8626278 10.1128/jb.178.6.1532-1538.1996 PMC177835

[52] Strous M, Van Gerven E, Kuenen JG, Jetten M. Effects of Aerobic and Microaerobic Conditions on Anaerobic Ammonium-Oxidizing (Anammox) Sludge. App Environ Microbiol. 1997;63: 2446–2448. 10.1128/aem.63.6.2446-2448.1997 PMC1389188 16535633

[53] Das A, Silaghi-Dumitrescu R, Ljungdahl LG, Kurtz DMJ. Cytochrome bd Oxidase, Oxidative Stress, and Dioxygen Tolerance of the Strictly Anaerobic Bacterium Moorella thermoacetica. J Bacteriol. 2005;187: 2020–2029. 15743950 10.1128/JB.187.6.2020-2029.2005 PMC1064043

[54] Schunck H, Lavik G, Desai DK, Großkopf T, Kalvelage T, Löscher CR, et al Giant Hydrogen Sulfide Plume in the Oxygen Minimum Zone off Peru Supports Chemolithoautotrophy. PLoS One. 2013;8: e68661 10.1371/journal.pone.0068661 23990875 PMC3749208

[55] Alldredge AL, Cohen Y. Can Microscale Chemical Patches Persist in the Sea? Microelectrode Study of Marine Snow, Fecal Pellets. Science. 1987;235: 689–691. 17833630 10.1126/science.235.4789.689

[56] Ploug H. Small-scale oxygen fluxes and remineralization in sinking aggregates. Limnol Ocean. 2001;46: 1624–1631.

[57] Shanks AL, Reeder ML. Reducing microzones and sulfide production in marine snow. Mar Ecol Progr Ser. 1993;96: 43–47.

[58] Ploug H, Kühl M, Buchholz-Cleven B, Jörgensen BB. Anoxic aggregates—an ephemeral phenomenon in the pelagic environment? Aquat Microb Ecol. 1997;13: 285–294.

[59] Klawonn I, Bonaglia S, Brüchert V, Ploug H. Aerobic and anaerobic nitrogen transformation processes in N2-fixing cyanobacterial aggregates. ISME J. 2015;9: 1456–66. 10.1038/ismej.2014.232 25575306 PMC4438332

[60] Stocker R. Marine Microbes See a Sea of Gradients. Science. 2012;388: 628–633. 10.1126/science.1208929 23118182

[61] Woebken D, Fuchs BA, Kuypers MAA, Amann R. Potential interactions of particle-associated anammox bacteria with bacterial and archaeal partners in the Namibian upwelling system. App Environ Microbiol. 2007;73: 4648–4657. 10.1128/AEM.02774-06 PMC1932835 17526789

[62] Ganesh S, Parris DJ, Delong EF, Fallon SJ. Metagenomic analysis of size-fractioned picoplankton in a marine oxygen minimum zone. ISME J. 2014;8: 187–211. 10.1038/ismej.2013.144 24030599 PMC3869020

[63] Dalsgaard T, Stewart FJ, Thamdrup B, De Brabandere L, Revsbech NP, Ulloa O, et al Oxygen at Nanomolar Levels Reversibly Suppresses Process Rates and Gene Expression in Anammox and Denitrification in the Oxygen Minimum Zone off Northern Chile. MBio. 2014;5: e01966–14. 10.1128/mBio.01966-14 25352619 PMC4217175

[64] Ward BB. How Nitrogen Is Lost. Science. 2013;341: 352–353. 10.1126/science.1240314 23888027

[65] Ward BB, Tuit CB, Jayakumar A, Rich JJ, Moffett J, Naqvi SWA. Organic carbon, and not copper, controls denitrification in oxygen minimum zones of the ocean. Deep Res I. 2008;55: 1672–1683.

[66] Levipan HA, Quinones RA, Urrutia H. A time series of prokaryote secondary production in the oxygen minimum zone of the Humboldt current system, off central Chile. Progr Ocean. 2007;75: 531–549.

[67] Gundersen K, Heldal M, Norland S, Purdie DA, Knap AH. Elemental C, N, and P cell content of individual bacteria collected at the Bermuda Atlantic Time-series Study (BATS) site. Limnol Ocean. 2002;47: 1525–1530.

[68] Fukuda R, Ogawa H, Nagat T, Koike I. Direct Determination of Carbon and Nitrogen Contents of Natural Bacterial Assemblages in Marine Environments. App Environm Microbiol. 1998;64: 3352–3358. 10.1128/aem.64.9.3352-3358.1998 PMC106732 9726882

[69] Landolfi A, Dietze H, Koeve W, Oschlies A. Overlooked runaway feedback in the marine nitrogen cycle: the vicious cycle. Biogeosciences. 2013;10: 1351–1363.

