# Supplementary material for: Aerobic Microbial Respiration In Oceanic Oxygen Minimum Zones
Source: PLoS One. 2015 Jul 20;10(7):e0133526. doi: 10.1371/journal.pone.0133526 (PMC4507870; doi:10.1371/journal.pone.0133526)
Supplement: S1 File — (PDF) [file pone.0133526.s001.pdf]

S1 Supplementary Methods

Modelling of oxygen consumption rates. Density was calculated using the data processing program SeaSoft (Sea-Bird Electronics). The stability of the water column was expressed using the Brunt-Väisälä frequency *N*, defined as:

$$N^2 = \frac{g}{\rho}\frac{\partial \rho}{\partial z}$$

where ρ is the water density, g is the gravitational acceleration and z is the water depth. The density gradient was calculated over 3-6 m bins. The turbulent diffusivity *Ez* was calculated according to Gregg et al. [1] from the Brunt-Väisälä-Frequency and the dissipation rate of turbulent kinetic energy ε:

$$Ez = \frac{\gamma \varepsilon}{N^2}$$

A mixing coefficient γ of 0.2 was applied. We used a mean ε of 1.85 x 10^{-9} W kg^{-1}. This value was measured by Gregg et al. [1] for the open ocean thermocline and was applied in several rate diffusion models [2] [3]. Concentration gradients for O_2 were calculated over 2-6 m bins. Oxygen fluxes at respective depths were calculated according to Fick's law:

$$J = -Ez\frac{\partial C}{\partial z}$$

O_2 consumption was determined from O_2 flux gradients, calculated over 1-4 m bins:

$$R = \frac{\partial J}{\partial z}$$

Processing of Peruvian OMZ metagenome data. A total of 1,204,437 raw reads were obtained for the metagenome samples from the Peruvian OMZ. Raw reads were clustered using Cd-hit [4] with a sequence identity threshold of 98% and a word length of 8. The ribosomal-gene cluster representative sequences were identified by BLASTn searches [5] against the SILVA database [6] (bit score cut off: 86). Of all sequences, 0.24% were of ribosomal gene origin and subsequently separated from non-ribosomal-gene cluster representative sequences using MEGAN [7]. The latter were compared against the non-redundant NCBI database using BLASTx (bit score cut off: 35) and scanned with profile hidden Markov models of the ModEnzA Enzyme Commission groups [8]. Of all non-ribosomal-gene sequences, 69.6% were identified as protein-coding; the remainder could not be assigned. Sequences, cluster sizes and cluster identification numbers as well as results from the BLAST searches and EC scans were added to a MySQL database for analysis [9,10]. For the functional (cytochrome oxidase type) and taxonomic assignment of the cluster representatives the top hit of each BLAST search was used.

Aggregate-size-dependent respiration rates. Diffusion-limited aerobic respiration (R) below a threshold O_2 concentration was estimated by rearranging the analytical solution for solute transport and reaction in a sphere [11]:

$$R = R_0 - R_0\left(1 - \frac{6 * C * D_{agg}}{R_0 * r_0^2}\right)^{\frac{3}{2}}$$

Here, R_0 is the non-limited O_2 consumption rate, C is the ambient O_2 concentration, D_{agg} is the diffusion coefficient inside the aggregate (1.3 x 10^{-9} m^2 s^{-1}), and r_0 is the aggregate diameter. For simplicity, the diffusive boundary layer around the aggregate was neglected and zero-order O_2 consumption was assumed. An empirically

determined relationship between aggregate diameter (in mm) and respiration rate (in nmol h^{-1}) in the Mauritanian upwelling region [12], with $R_0 = 1.8\ d^{1.8}$, was used to calculate O_2 consumption as a function of O_2 concentration for particles of 0.01 - 10 mm in diameter. To account for the somewhat lower incubation temperatures in our study ($\Delta O_2 \approx 5$ °C, Supplementary Table 1 and 2), O_2 consumption rates were corrected using a temperature coefficient (Q_{10}) of 2 [13].

Stoichiometries used to calculate NH_4^+ budgets for the upper Namibian and Peruvian OMZs:

Aerobic remineralization: $(CH_2O)_{106}(NH_3)_{16}H_3PO_4 + 106\ O_2 + 16\ H^+$

$\rightarrow 106\ CO_2 + 16\ NH_4^+ + 106\ H_2O + H_3PO_4$

Nitrification:

NH_4^+ oxidation: $NH_3 + 1.5\ O_2 \rightarrow NO_2^- + H^+ + H_2O$

NO_2^- oxidation: $NO_2^- + 0.5\ O_2 \rightarrow NO_3^-$

NO_3^- reduction to NO_2^-: $(CH_2O)_{106}(NH_3)_{16}H_3PO_4 + 212\ NO_3^- + 16\ H^+$

$\rightarrow 106\ CO_2 + 16\ NH_4^+ + 212\ NO_2^- + 106\ H_2O + H_3PO_4$

DNRA: $(CH_2O)_{106}(NH_3)_{16}H_3PO_4 + 53\ NO_3^- + 122\ H^+$

$\rightarrow 106\ CO_2 + 69\ NH_4^+ + 53\ H_2O + H_3PO_4$

Anammox: $NH_4^+ + NO_2^- \rightarrow N_2 + 2\ H_2O$

References

1. Gregg MC, D'Asaro EA, Shay TJ, Larson N. Observations of Persistent Mixing and Near-Inertial Internal Waves. J Phys Ocean. 1986;16: 856–885.

2. Fennel W. A model of the yearly cycle of nutrients and plankton in the Baltic Sea. J Mar Sci. 1995;6: 313–329.

3. Lam P, Jensen MM, Lavik G, McGinnis DF, Muller B, Schubert CJ, et al. Linking crenarchaeal and bacterial nitrification to anammox in the Black Sea. PNAS. 2007;104: 7104–7109.

4. Li W, Godzik A. Cd-hit: a fast program for clustering and comparing large sets of protein or nucleotide sequences. Bioinformatics. 2006;22: 1658–1659.

5. Altschul S, Gish W, Miller W, Myers E, Lipman D. Basic Local Alignment Search Tool. J Mol Biol. 1990;215: 403–410.

6. Prüsse E, Quast C, Knittel K, Fuchs B, W L, Peplies J, et al. SILVA: a comprehensive online resource for quality checked and aligned ribosomal RNA sequence data compatible with ARB. Nucleic Acids Res. 2007;35: 7188–7196.

7. Huson D, Auch A, Qi J, Schuster S. MEGAN analysis of metagenomic data. Genome Res. 2007;17: 377–386.

8. Desai D, Nandi S, Srivastata P, Lynn A. ModEnza: Accurate Identification of Metabolic Enzymes Using Function Specific Profile HMMs with Optimized Discrimination Threshold and Modified Emission Probabilities. Adv Bioinformatics. 2011; 743782.

9. Desai DK, Schunck H, Löser JW, LaRoche J. Fragment Recruitment on Metabolic Pathways: comparative metabolic profiling of metagenomes and metatranscriptomes. Bioinformatics. 2013;29: 790–791.

10. Schunck H, Lavik G, Desai DK, Großkopf T, Kalvelage T, Löscher CR, et al. Giant Hydrogen Sulfide Plume in the Oxygen Minimum Zone off Peru Supports Chemolithoautotrophy. PLoS One. 2013;8: e68661.

11. Ploug H, Kühl M, Buchholz-Cleven B, Jörgensen BB. Anoxic aggregates - an ephemeral phenomenon in the pelagic environment? Aquat Microb Ecol. 1997;13: 285–294.

12. Iversen MH, Nowald N, Ploug H, Jackson GA, Fischer G. High resolution profiles of vertical particulate organic matter export off Cape Blanc, Mauritania: Degradation processes and ballasting effects. Deep Res I. 2010;57: 771–784.

13. Thamdrup B, Hansen JW, Jørgensen BB. Temperature dependence of aerobic respiration in a coastal sediment. FEMS Microbiol Ecol. 1998;25: 189–200.
