# Supplementary material for: Aerobic Microbial Respiration In Oceanic Oxygen Minimum Zones
Source: PLoS One. 2015 Jul 20;10(7):e0133526. doi: 10.1371/journal.pone.0133526 (PMC4507870; doi:10.1371/journal.pone.0133526)
Supplement: S1 Fig — (PDF) [file pone.0133526.s002.pdf]

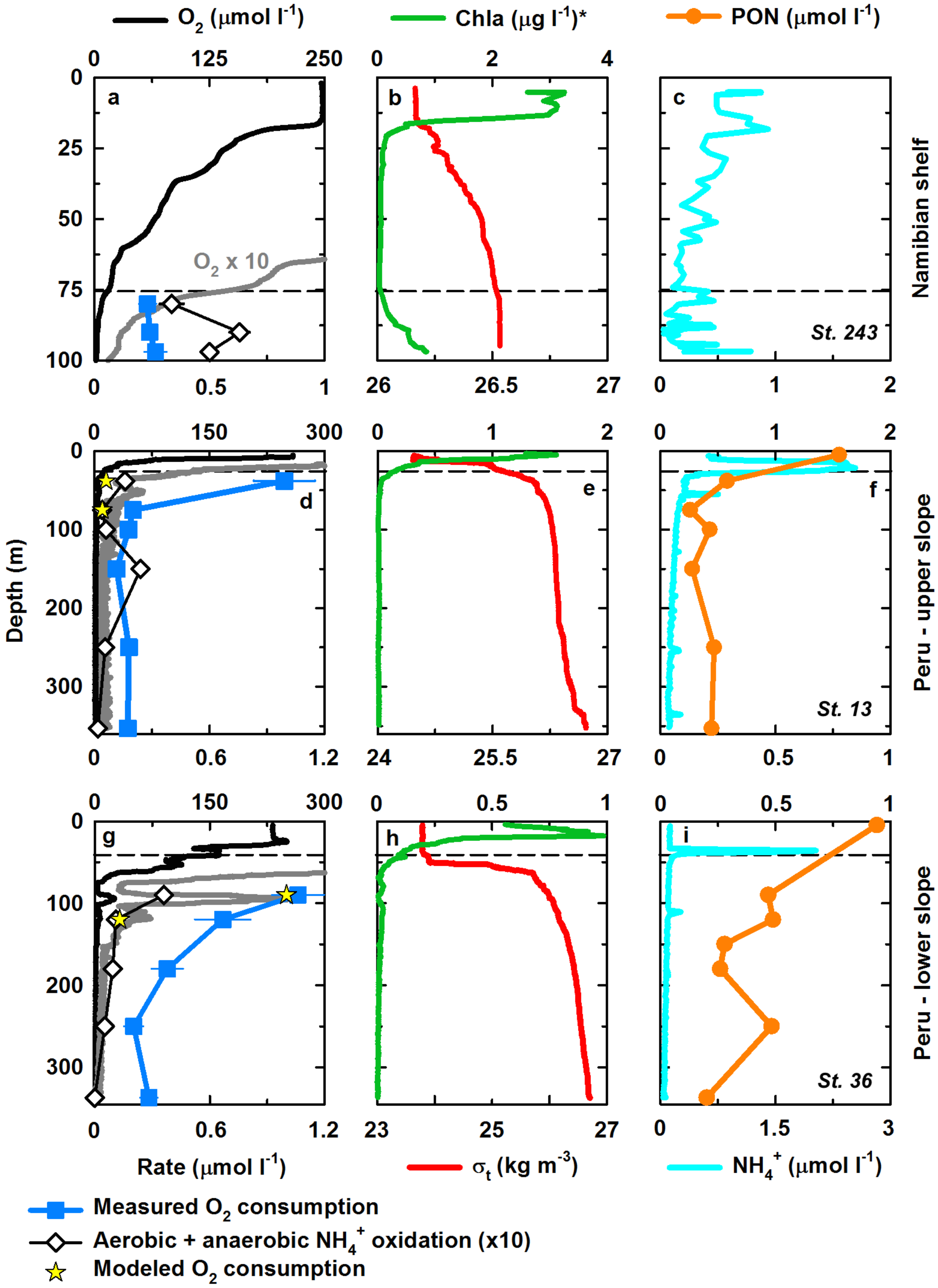

S1 Figure. Physicochemical zonation and rates of microbial respiration in the OMZs off Namibia and Peru. a-c, Namibian shelf (station 243, 103 m). **d-f,** Upper Peruvian continental slope (station 13, 356 m). **g-i,** Lower Peruvian continental slope (station 36, 2845 m). Dashed lines indicate the upper OMZ boundary (O_2 ≤15 µmol l^{-1}). Rates of aerobic + anaerobic NH_4^+ oxidation are 10 x magnified. *Chlorophyll a concentrations in panel **b** in relative units.
