# Supplementary material for: Aerobic Microbial Respiration In Oceanic Oxygen Minimum Zones
Source: PLoS One. 2015 Jul 20;10(7):e0133526. doi: 10.1371/journal.pone.0133526 (PMC4507870; doi:10.1371/journal.pone.0133526)
Supplement: S2 Fig — (PDF) [file pone.0133526.s003.pdf]

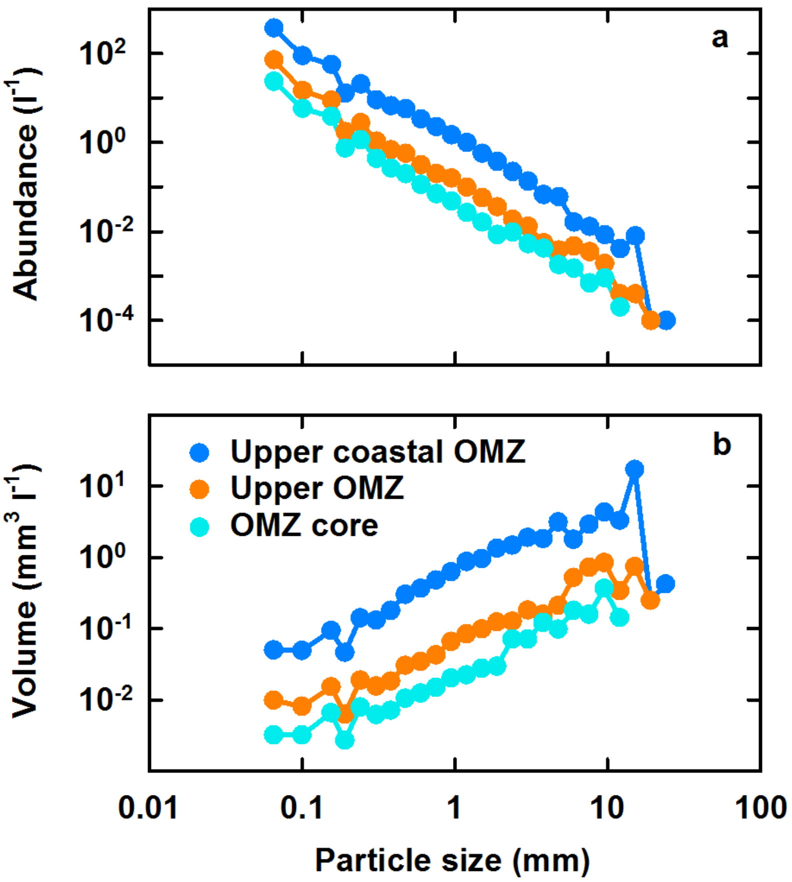

S2 Figure. Particle size distributions in the South Pacific OMZ. Particle abundance (a) and volume (b) for particle size classes between 0.06 and 26.8 mm (ESD) based on 138 Underwater Vision Profiler (UVP5) deployments off central Peru during cruise M93. Abundance and volume are means of 20-m bins centred on 30 - 70 m (upper coastal OMZ), as well as 70 - 90 m (upper OMZ) and 350 m (OMZ core) at water depths ≥360 m. Particle size classes >0.5 mm include both detritus and zooplankton, at a detritus/zooplankton ratio of on average 9:1.
