# Supplementary material for: Aerobic Microbial Respiration In Oceanic Oxygen Minimum Zones
Source: PLoS One. 2015 Jul 20;10(7):e0133526. doi: 10.1371/journal.pone.0133526 (PMC4507870; doi:10.1371/journal.pone.0133526)
Supplement: S1 Table — (PDF) [file pone.0133526.s004.pdf]

S1 Table. Oxygen consumption rates in the OMZs off Namibia and Peru. Adjusted $^{18-18}O_2$ concentrations are given in the method section. The upper Peruvian OMZ is here loosely defined by O_2 ≥0.5 µmol l^{-1}, except for station 805 where higher O_2 concentrations persisted throughout the OMZ. SE = standard error; n.a. = not available; b.d. = below detection;* STOX sensor measurements.

Cruise	Station	Lat (°S)	Lon (°E)	Water depth (m)	Zone	Sampled depth (m)	*In-situ* T (°C)	*In-situ* O_2 (µM)	Respiration rate (µM O_2/d)	± SE
M76-2 (Namibia)	225	19.02	12.24	123	OMZ	85	14.2	4.4	0.45	0.06
						90	13.9	4.1	0.27	0.05
						110	13.5	0.3	0.29	0.07
					BBL	120.94	n.a.	n.a.	0.26	0.03
						121.88	n.a.	n.a.	0.23	0.03
						122.7	n.a.	n.a.	0.22	0.05
	231	21.00	13.25	123	OMZ	105	13.5	8.1	0.14	0.04
						115	13.4	5.9	0.16	0.03
						118	13.4	5.8	0.14	0.02
					BBL	120.94	n.a.	n.a.	0.20	0.03
						121.88	n.a.	n.a.	0.28	0.04
						122.7	n.a.	n.a.	0.35	0.09
	243	22.10	13.87	103	OMZ	80	13.3	7.6	0.23	0.04
						90	13.3	2.9	0.24	0.03
						97	13.3	2.4	0.26	0.05
					BBL	100.94	n.a.	n.a.	0.19	0.06
						101.88	n.a.	n.a.	0.44	0.08
						102.7	n.a.	n.a.	0.23	0.05
	252	23.00	14.23	111	OMZ	76	12.8	1.1*	0.45	0.04
						95	12.6	0.0*	0.92	0.31
						105	12.6	0.0*	1.62	0.20
					BBL	108.94	n.a.	n.a.	1.47	0.18
						109.88	n.a.	n.a.	1.50	0.32
						110.7	n.a.	n.a.	1.26	0.26
M77-3 (Peru)	805	6.00	-81.36	999	upper OMZ	62	14.8	7.5	0.54	0.06
						69	14.7	1.5	0.34	0.08
					OMZ core	197	13.4	1.5	0.26	0.07
						345	10.6	0.6	0.35	0.06
	807	10.00	-78.38	115	upper OMZ	15	16.3	~20	3.14	0.10
						20	16.1	0.6	0.49	0.05
					OMZ core	40	14.9	0.0	0.21	0.02
						60	14.6	0.0	0.38	0.05
						80	14.5	0.0	0.28	0.02
						110	14.5	0.0	0.32	0.06
	811	10.00	-78.97	145	upper OMZ	54	16.0	4.9	0.61	0.15
					OMZ core	80	14.9	0.0	b.d.	-
						100	14.4	0.0	b.d.	-
						120	14.2	0.0	b.d.	-
						140	14.1	0.0	0.18	0.04
	3	10.00	-81.50	4697	upper OMZ	52	17.3	4.0	1.19	0.11
						70	16.0	1.5	0.96	0.14
					OMZ core	95	15.0	0.2	0.39	0.05
						150	14.2	0.2	0.27	0.07
						300	12.4	0.0	b.d.	-
						365	11.1	0.0	b.d.	-
	5	10.00	-84.00	4525	upper OMZ	75	15.9	2.6	0.73	0.07
					OMZ core	110	14.3	0.4	0.35	0.05
						150	13.5	0.1	0.15	0.02
						200	12.9	0.0	0.24	0.05
						275	12.1	0.0	b.d.	-
						362	11.1	0.0	b.d.	-
	13	12.03	-77.79	356	upper OMZ	38	15.7	3.4*	0.99	0.16
					OMZ core	75	14.6	0.0*	0.20	0.04
						100	14.3	0.0*	0.18	0.04
						150	14.0	0.0*	0.12	0.01
						250	13.4	0.1*	0.18	0.02
						353	11.7	0.0*	0.17	0.02
	36	16.00	-75.00	2845	upper OMZ	90	14.6	1.5*	1.06	0.14
						120	13.5	1.2*	0.67	0.15
					OMZ core	180	12.8	0.0*	0.38	0.08
						250	12.0	0.0*	0.20	0.05
						337	10.9	0.0*	0.28	0.05
