# Supplementary material for: Aerobic Microbial Respiration In Oceanic Oxygen Minimum Zones
Source: PLoS One. 2015 Jul 20;10(7):e0133526. doi: 10.1371/journal.pone.0133526 (PMC4507870; doi:10.1371/journal.pone.0133526)
Supplement: S2 Table — (PDF) [file pone.0133526.s005.pdf]

S2 Table. O_2 sensitivity assays in the OMZs off Namibia and Peru. Adjusted O_2 concentrations (initial $^{18\text{-}18}O_2$ + $^{16\text{-}16}O_2$ background) were determined using either STOX sensors or Clark-type microsensors. Higher O_2 treatments often showed O_2 consumption rates tailing off between 12 - 24 h of incubation and only samples falling within the initial linear phase were considered for rate calculations, hence the larger uncertainties for these experiments. SE = standard error; b.d. = below detection; * STOX sensor measurements.

Cruise	Station	Lat (°S)	Lon (°E)	Water depth (m)	Sampled depth (m)	*In-situ* T (°C)	*In-situ* O_2 (µM)	Adjusted O_2 (µM)	Respiration rate (µM O_2/d)	± SE
**M76-2 (Namibia)**	225	19.02	12.24	123	90	13.9	4.1	2.6	0.27	0.05
								6.1	1.67	0.18
								8.8	4.71	0.91
								12.5	6.41	0.97
					110	13.5	0.3	2.5	0.29	0.07
								5.6	3.93	0.51
								10.4	6.06	1.35
								15.0	8.35	0.54
**M77-3 (Peru)**	13	12.03	-77.79	356	75	14.6	0.0*	0.5	b.d.	-
								1.2	0.09	0.01
								3.2	0.20	0.04
								8.3	0.48	0.09
								19.2	1.46	0.28
					353	11.7	0.0*	0.7	b.d.	-
								1.6	0.17	0.02
								3.6	0.30	0.03
								8.0	0.45	0.05
								18.4	0.90	0.09
	28	16.00	-77.00	2352	357	11.7	0.0*	1.0	b.d.	-
								1.5	0.09	0.01
								3.4	0.14	0.01
								7.4	0.56	0.09
								18.5	1.19	0.24
