# Supplementary material for: Aerobic Microbial Respiration In Oceanic Oxygen Minimum Zones
Source: PLoS One. 2015 Jul 20;10(7):e0133526. doi: 10.1371/journal.pone.0133526 (PMC4507870; doi:10.1371/journal.pone.0133526)
Supplement: S3 Table — (PDF) [file pone.0133526.s006.pdf]

S3 Table. Abundance and taxonomic assignment of BLAST hits for low-affinity and high-affinity (bd and cbb_3) cytochrome oxidases of metagenomes and metatranscriptomes from the South Pacific OMZ.

Peruvian OMZ Metagenome (M77-3, station 3 10.00°S/81.50°W)		Low-affinity cyt oxidase			Cyt bd oxidase			Cyt cbb_3 oxidase			All
		DNA hit count	% of cyt ox hits	% of total hits	DNA hit count	% of cyt bd hits	% of total hits	DNA hit count	% of cbb_3 hits	% of total hits	% of all cyt hits
	Alphaproteobacteria	111	27.8	0.0605	0	0.0	0.0000	21	52.5	0.0115	28.9
	Betaproteobacteria	21	5.3	0.0115	2	12.5	0.0011	0	0.0	0.0000	5.0
	Gammaproteobacteria	61	15.3	0.0333	7	43.8	0.0038	13	32.5	0.0071	17.8
	Deltaproteobacteria	7	1.8	0.0038	0	0.0	0.0000	0	0.0	0.0000	1.5
	Epsilonproteobacteria	1	0.3	0.0005	0	0.0	0.0000	5	12.5	0.0027	1.3
	Archaea	42	10.5	0.0229	0	0.0	0.0000	0	0.0	0.0000	9.2
	Cyanobacteria	22	5.5	0.0120	0	0.0	0.0000	0	0.0	0.0000	4.8
	Chlamydiae/Verrucom.	36	9.0	0.0196	0	0.0	0.0000	0	0.0	0.0000	7.9
	Bacteriodetes/Chlorobi	37	9.3	0.0202	0	0.0	0.0000	1	2.5	0.0005	8.3
50m	Gemmatimonadetes	5	1.3	0.0027	0	0.0	0.0000	0	0.0	0.0000	1.1
	Planctomycetes	5	1.3	0.0027	5	31.3	0.0027	0	0.0	0.0000	2.2
	Firmicutes	3	0.8	0.0016	0	0.0	0.0000	0	0.0	0.0000	0.7
	Aquificae	1	0.3	0.0005	0	0.0	0.0000	0	0.0	0.0000	0.2
	Actinobacteria	17	4.3	0.0093	0	0.0	0.0000	0	0.0	0.0000	3.7
	Chloroflexi	7	1.8	0.0038	0	0.0	0.0000	0	0.0	0.0000	1.5
	Nitrospirae	0	0.0	0.0000	2	12.5	0.0011	0	0.0	0.0000	0.4
	Unknown/unclassified	24	6.0	0.0131	0	0.0	0.0000	0	0.0	0.0000	5.3
	∑	400	100.0	0.2181	16	100.0	0.0087	40	100.0	0.0218	100.0
	Alphaproteobacteria	117	31.9	0.0612	7	18.9	0.0037	48	60.0	0.0251	35.5
	Betaproteobacteria	19	5.2	0.0099	1	2.7	0.0005	7	8.8	0.0037	5.6
	Gammaproteobacteria	52	14.2	0.0272	0	0.0	0.0000	12	15.0	0.0063	13.2
	Deltaproteobacteria	26	7.1	0.0136	0	0.0	0.0000	0	0.0	0.0000	5.4
	Epsilonproteobacteria	0	0.0	0.0000	0	0.0	0.0000	2	2.5	0.0010	0.4
	Archaea	11	3.0	0.0058	0	0.0	0.0000	0	0.0	0.0000	2.3
	Cyanobacteria	11	3.0	0.0058	0	0.0	0.0000	0	0.0	0.0000	2.3
	Chlamydiae/Verrucom.	41	11.2	0.0214	0	0.0	0.0000	0	0.0	0.0000	8.5
	Bacteriodetes/Chlorobi	17	4.6	0.0089	0	0.0	0.0000	4	5.0	0.0021	4.3
90m	Gemmatimonadetes	3	0.8	0.0016	0	0.0	0.0000	0	0.0	0.0000	0.6
	Planctomycetes	5	1.4	0.0026	18	48.6	0.0094	7	8.8	0.0037	6.2
	Firmicutes	9	2.5	0.0047	0	0.0	0.0000	0	0.0	0.0000	1.9
	Aquificae	14	3.8	0.0073	0	0.0	0.0000	0	0.0	0.0000	2.9
	Fibrobacteres	3	0.8	0.0016	0	0.0	0.0000	0	0.0	0.0000	0.6
	Actinobacteria	10	2.7	0.0052	0	0.0	0.0000	0	0.0	0.0000	2.1
	Chloroflexi	7	1.9	0.0037	0	0.0	0.0000	0	0.0	0.0000	1.4
	Nitrospirae	0	0.0	0.0000	11	29.7	0.0058	0	0.0	0.0000	2.3
	Unknown/unclassified	22	6.0	0.0115	0	0.0	0.0000	0	0.0	0.0000	4.5
	∑	367	100.0	0.1919	37	100.0	0.0193	80	100.0	0.0418	100.0
	Alphaproteobacteria	183	35.5	0.0830	19	28.4	0.0086	43	43.4	0.0195	35.9
	Betaproteobacteria	39	7.6	0.0177	1	1.5	0.0005	5	5.1	0.0023	6.6
	Gammaproteobacteria	105	20.3	0.0476	4	6.0	0.0018	17	17.2	0.0077	18.5
	Deltaproteobacteria	48	9.3	0.0218	0	0.0	0.0000	0	0.0	0.0000	7.0
	Epsilonproteobacteria	0	0.0	0.0000	0	0.0	0.0000	2	2.0	0.0009	0.3
	Archaea	6	1.2	0.0027	0	0.0	0.0000	0	0.0	0.0000	0.9
	Cyanobacteria	8	1.6	0.0036	0	0.0	0.0000	0	0.0	0.0000	1.2
	Chlamydiae/Verrucom.	35	6.8	0.0159	0	0.0	0.0000	1	1.0	0.0005	5.3
	Bacteriodetes/Chlorobi	21	4.1	0.0095	0	0.0	0.0000	3	3.0	0.0014	3.5
150m	Gemmatimonadetes	3	0.6	0.0014	0	0.0	0.0000	0	0.0	0.0000	0.4
	Planctomycetes	11	2.1	0.0050	33	49.3	0.0150	28	28.3	0.0127	10.6
	Firmicutes	1	0.2	0.0005	0	0.0	0.0000	0	0.0	0.0000	0.1
	Aquificae	18	3.5	0.0082	0	0.0	0.0000	0	0.0	0.0000	2.6
	Fibrobacteres	11	2.1	0.0050	0	0.0	0.0000	0	0.0	0.0000	1.6
	Actinobacteria	4	0.8	0.0018	0	0.0	0.0000	0	0.0	0.0000	0.6
	Chloroflexi	3	0.6	0.0014	0	0.0	0.0000	0	0.0	0.0000	0.4
	Nitrospirae	0	0.0	0.0000	7	10.4	0.0032	0	0.0	0.0000	1.0
	Unknown/unclassified	20	3.9	0.0091	3	4.5	0.0014	0	0.0	0.0000	3.4
	∑	516	100.0	0.2339	67	100.0	0.0304	99	100.0	0.0449	100.0

Peruvian OMZ Metagenome (M77-3, station 3 10.00°S/81.50°W)		Low-affinity cyt oxidase			Cyt bd oxidase			Cyt cbb$_3$ oxidase			All
		DNA hit count	% of cyt ox hits	% of total hits	DNA hit count	% of cyt bd hits	% of total hits	DNA hit count	% of cbb3 hits	% of total hits	% of all cyt hits
380m	Alphaproteobacteria	276	42.7	0.1134	26	37.1	0.0107	87	73.7	0.0357	46.6
	Betaproteobacteria	60	9.3	0.0247	0	0.0	0.0000	2	1.7	0.0008	7.4
	Gammaproteobacteria	145	22.4	0.0596	2	2.9	0.0008	11	9.3	0.0045	18.9
	Deltaproteobacteria	34	5.3	0.0140	0	0.0	0.0000	1	0.8	0.0004	4.2
	Archaea	4	0.6	0.0016	0	0.0	0.0000	0	0.0	0.0000	0.5
	Cyanobacteria	4	0.6	0.0016	0	0.0	0.0000	0	0.0	0.0000	0.5
	Chlamydiae/Verrucom.	38	5.9	0.0156	2	2.9	0.0008	1	0.8	0.0004	4.9
	Bacteriodetes/Chlorobi	18	2.8	0.0074	0	0.0	0.0000	1	0.8	0.0004	2.3
	Gemmatimonadetes	2	0.3	0.0008	0	0.0	0.0000	0	0.0	0.0000	0.2
	Planctomycetes	12	1.9	0.0049	26	37.1	0.0107	15	12.7	0.0062	6.4
	Aquificae	22	3.4	0.0090	0	0.0	0.0000	0	0.0	0.0000	2.6
	Fibrobacteres	1	0.2	0.0004	0	0.0	0.0000	0	0.0	0.0000	0.1
	Actinobacteria	6	0.9	0.0025	0	0.0	0.0000	0	0.0	0.0000	0.7
	Chloroflexi	2	0.3	0.0008	0	0.0	0.0000	0	0.0	0.0000	0.2
	Nitrospirae	0	0.0	0.0000	10	14.3	0.0041	0	0.0	0.0000	1.2
	Unknown/unclassified	22	3.4	0.0090	4	5.7	0.0016	0	0.0	0.0000	3.1
	∑	646	100.0	0.2654	70	100.0	0.0288	118	100.0	0.0485	100.0

Chilean OMZ Metagenome (MOOMZ-1, station 3 20.12°S/70.38°W)		Low-affinity cyt oxidase			Cyt bd oxidase			Cyt cbb$_3$ oxidase			All
		DNA hit count	% of cyt ox hits	% of total hits	DNA hit count	% of cyt bd hits	% of total hits	DNA hit count	% of cbb3 hits	% of total hits	% of all cyt hits
50m	Alphaproteobacteria	173	32.9	0.0846	1	50.0	0.0005	25	80.9	0.0124	35.7
	Betaproteobacteria	10	1.9	0.0048	0	0.0	0.0000	3	9.6	0.0015	2.3
	Gammaproteobacteria	74	14.0	0.0360	0	0.0	0.0000	2	6.4	0.0010	13.5
	Deltaproteobacteria	12	2.3	0.0059	0	0.0	0.0000	0	0.0	0.0000	2.1
	Epsilonproteobacteria	0	0.0	0.0000	0	0.0	0.0000	1	3.2	0.0005	0.2
	Archaea	41	7.7	0.0198	0	0.0	0.0000	0	0.0	0.0000	7.2
	Cyanobacteria	18	3.4	0.0088	0	0.0	0.0000	0	0.0	0.0000	3.2
	Chlamydiae/Verrucom.	66	12.4	0.0320	0	0.0	0.0000	0	0.0	0.0000	11.7
	Bacteriodetes/Chlorobi	46	8.7	0.0223	0	0.0	0.0000	0	0.0	0.0000	8.1
	Gemmatimonadetes	4	0.8	0.0020	0	0.0	0.0000	0	0.0	0.0000	0.7
	Planctomycetes	13	2.5	0.0063	1	50.0	0.0005	0	0.0	0.0000	2.5
	Firmicutes	7	1.3	0.0034	0	0.0	0.0000	0	0.0	0.0000	1.2
	Actinobacteria	10	1.9	0.0049	0	0.0	0.0000	0	0.0	0.0000	1.8
	Chloroflexi	20	3.8	0.0098	0	0.0	0.0000	0	0.0	0.0000	3.6
	Deinococcus-Thermus	3	0.6	0.0015	0	0.0	0.0000	0	0.0	0.0000	0.5
	Acidobacteria	4	0.8	0.0020	0	0.0	0.0000	0	0.0	0.0000	0.7
	Unknown/unclassified	27	5.2	0.0133	0	0.0	0.0000	0	0.0	0.0000	4.9
	∑	527	100.0	0.2572	2	100.0	0.0010	31	100.0	0.0153	100.0
85m	Alphaproteobacteria	355	34.3	0.1041	0	0.0	0.0000	91	66.8	0.0266	37.7
	Betaproteobacteria	72	7.0	0.0212	0	0.0	0.0000	18	13.2	0.0052	7.6
	Gammaproteobacteria	140	13.6	0.0411	1	4.8	0.0001	11	8.3	0.0033	12.9
	Deltaproteobacteria	20	2.0	0.0060	0	0.0	0.0000	0	0.0	0.0000	1.7
	Epsilonproteobacteria	0	0.0	0.0000	0	0.0	0.0000	8	5.9	0.0023	0.7
	Archaea	98	9.5	0.0288	0	0.0	0.0000	0	0.0	0.0000	8.3
	Cyanobacteria	16	1.5	0.0045	0	0.0	0.0000	0	0.0	0.0000	1.3
	Chlamydiae/Verrucom.	133	12.8	0.0390	0	0.0	0.0000	2	1.5	0.0006	11.4
	Bacteriodetes/Chlorobi	50	4.8	0.0146	0	0.0	0.0000	3	2.2	0.0009	4.5
	Gemmatimonadetes	5	0.5	0.0016	0	0.0	0.0000	0	0.0	0.0000	0.5
	Planctomycetes	23	2.3	0.0068	10	95.2	0.0029	3	2.2	0.0009	3.1
	Firmicutes	17	1.7	0.0051	0	0.0	0.0000	0	0.0	0.0000	1.5
	Aquificae	13	1.3	0.0038	0	0.0	0.0000	0	0.0	0.0000	1.1
	Actinobacteria	8	0.8	0.0023	0	0.0	0.0000	0	0.0	0.0000	0.7
	Chloroflexi	30	2.8	0.0086	0	0.0	0.0000	0	0.0	0.0000	2.5
	Deinococcus-Thermus	4	0.4	0.0012	0	0.0	0.0000	0	0.0	0.0000	0.3

Chilean OMZ Metagenome (MOOMZ-1, station 3 20.12°S/70.38°W)		Low-affinity cyt oxidase			Cyt bd oxidase			Cyt cbb_3 oxidase			All
		DNA hit count	% of cyt ox hits	% of total hits	DNA hit count	% of cyt bd hits	% of total hits	DNA hit count	% of cbb3 hits	% of total hits	% of all cyt hits
85m	Acidobacteria	8	0.7	0.0022	0	0.0	0.0000	0	0.0	0.0000	0.6
	Lentisphaera	5	0.5	0.0015	0	0.0	0.0000	0	0.0	0.0000	0.4
	Spirochaetes	5	0.4	0.0013	0	0.0	0.0000	0	0.0	0.0000	0.4
	Unknown/unclassified	34	3.3	0.0099	0	0.0	0.0000	0	0.0	0.0000	2.9
	∑	1036	100.0	0.3036	11	100.0	0.0031	136	100.0	0.0399	100.0
110m	Alphaproteobacteria	265	31.4	0.1231	0	0.0	0.0000	33	49.8	0.0155	31.9
	Betaproteobacteria	60	7.1	0.0277	0	0.0	0.0000	10	14.2	0.0044	7.4
	Gammaproteobacteria	138	16.3	0.0639	2	8.3	0.0009	9	13.5	0.0042	15.9
	Deltaproteobacteria	37	4.3	0.0170	0	0.0	0.0000	0	0.0	0.0000	3.9
	Epsilonproteobacteria	0	0.0	0.0000	0	0.0	0.0000	1	1.5	0.0005	0.1
	Archaea	44	5.2	0.0204	0	0.0	0.0000	0	0.0	0.0000	4.7
	Cyanobacteria	16	1.9	0.0076	0	0.0	0.0000	0	0.0	0.0000	1.7
	Chlamydiae/Verrucom.	109	13.0	0.0508	0	0.0	0.0000	1	1.5	0.0005	11.8
	Bacteriodetes/Chlorobi	28	3.3	0.0130	0	0.0	0.0000	5	7.5	0.0023	3.5
	Gemmatimonadetes	6	0.7	0.0026	0	0.0	0.0000	0	0.0	0.0000	0.6
	Planctomycetes	24	2.8	0.0112	22	91.7	0.0102	8	12.0	0.0037	5.8
	Firmicutes	13	1.5	0.0058	0	0.0	0.0000	0	0.0	0.0000	1.3
	Aquificae	10	1.2	0.0046	0	0.0	0.0000	0	0.0	0.0000	1.1
	Actinobacteria	11	1.2	0.0049	0	0.0	0.0000	0	0.0	0.0000	1.1
	Chloroflexi	30	3.5	0.0138	0	0.0	0.0000	0	0.0	0.0000	3.2
	Deinococcus-Thermus	7	0.8	0.0033	0	0.0	0.0000	0	0.0	0.0000	0.7
	Acidobacteria	5	0.6	0.0023	0	0.0	0.0000	0	0.0	0.0000	0.5
	Lentisphaera	1	0.1	0.0005	0	0.0	0.0000	0	0.0	0.0000	0.1
	Spirochaetes	2	0.2	0.0007	0	0.0	0.0000	0	0.0	0.0000	0.2
	Unknown/unclassified	41	4.8	0.0190	0	0.0	0.0000	0	0.0	0.0000	4.4
	∑	844	100.0	0.3921	24	100.0	0.0112	67	100.0	0.0310	100.0
200m	Alphaproteobacteria	492	45.8	0.1791	0	0.0	0.0000	42	41.8	0.0153	43.8
	Betaproteobacteria	86	8.0	0.0314	1	2.9	0.0005	3	3.0	0.0011	7.4
	Gammaproteobacteria	170	15.9	0.0620	3	7.0	0.0012	20	19.9	0.0073	15.9
	Deltaproteobacteria	22	2.0	0.0080	0	0.0	0.0000	2	2.0	0.0007	2.0
	Archaea	4	0.4	0.0015	0	0.0	0.0000	0	0.0	0.0000	0.3
	Cyanobacteria	9	0.9	0.0033	0	0.0	0.0000	0	0.0	0.0000	0.8
	Chlamydiae/Verrucom.	93	8.7	0.0339	0	0.0	0.0000	0	0.0	0.0000	7.6
	Bacteriodetes/Chlorobi	24	2.2	0.0086	0	0.0	0.0000	1	1.0	0.0004	2.0
	Gemmatimonadetes	3	0.3	0.0011	0	0.0	0.0000	0	0.0	0.0000	0.2
	Planctomycetes	51	4.8	0.0186	41	90.1	0.0149	32	31.3	0.0115	10.1
	Firmicutes	6	0.6	0.0022	0	0.0	0.0000	0	0.0	0.0000	0.5
	Aquificae	28	2.6	0.0102	0	0.0	0.0000	0	0.0	0.0000	2.3
	Actinobacteria	3	0.2	0.0009	0	0.0	0.0000	0	0.0	0.0000	0.2
	Chloroflexi	18	1.7	0.0066	0	0.0	0.0000	0	0.0	0.0000	1.5
	Deinococcus-Thermus	9	0.8	0.0033	0	0.0	0.0000	0	0.0	0.0000	0.7
	Acidobacteria	4	0.4	0.0015	0	0.0	0.0000	0	0.0	0.0000	0.3
	Lentisphaera	2	0.2	0.0007	0	0.0	0.0000	1	1.0	0.0004	0.2
	Deferribacteres	1	0.1	0.0004	0	0.0	0.0000	0	0.0	0.0000	0.1
	Unknown/unclassified	49	4.6	0.0180	0	0.0	0.0000	0	0.0	0.0000	4.1
	∑	1073	100.0	0.3911	46	100.0	0.0166	101	100.0	0.0366	100.0

Chilean OMZ Metatranscriptome (MOOMZ-1, station 3 20.12°S/70.38°W)		Low-affinity cyt oxidase			Cyt bd oxidase			Cyt cbb$_3$ oxidase			All
		RNA hit count	% of cyt ox hits	% of total hits	RNA hit count	% of cyt bd hits	% of total hits	RNA hit count	% of cbb3 hits	% of total hits	% of all cyt hits
50m	Alphaproteobacteria	29	24.3	0.0426	0	0.0	0.0000	0	0.0	0.0000	21.8
	Betaproteobacteria	1	0.8	0.0005	0	0.0	0.0000	1	100.0	0.0005	1.5
	Gammaproteobacteria	23	19.3	0.0151	0	0.0	0.0000	0	0.0	0.0000	17.3
	Archaea	22	18.5	0.0141	0	0.0	0.0000	0	0.0	0.0000	16.5
	Cyanobacteria	5	4.2	0.0020	0	0.0	0.0000	0	0.0	0.0000	3.8
	Chlamydiae/Verrucom.	26	21.9	0.0105	0	0.0	0.0000	0	0.0	0.0000	19.6
	Gemmatimonadetes	3	2.5	0.0020	0	0.0	0.0000	0	0.0	0.0000	2.3
	Planctomycetes	1	0.8	0.0015	13	100.0	0.0005	0	0.0	0.0000	10.5
	Chloroflexi	2	1.7	0.0029	0	0.0	0.0000	0	0.0	0.0000	1.5
	Deinococcus-Thermus	1	0.8	0.0010	0	0.0	0.0000	0	0.0	0.0000	0.8
	Unknown/unclassified	6	5.0	0.0074	0	0.0	0.0000	0	0.0	0.0000	4.5
	∑	119	100.0	0.0996	13	100.0	0.0005	1	100.0	0.0005	100.0
85m	Alphaproteobacteria	11	26.8	0.0255	0	0.0	0.0000	1	100.0	0.0060	24.9
	Betaproteobacteria	5	12.7	0.0045	0	0.0	0.0000	0	0.0	0.0000	10.8
	Gammaproteobacteria	9	23.6	0.0136	0	0.0	0.0000	0	0.0	0.0000	20.0
	Archaea	2	5.1	0.0141	0	0.0	0.0000	0	0.0	0.0000	4.3
	Chlamydiae/Verrucom.	5	12.7	0.0140	0	0.0	0.0000	0	0.0	0.0000	10.8
	Bacteriodetes/Chlorobi	1	2.5	0.0015	0	0.0	0.0000	0	0.0	0.0000	2.2
	Planctomycetes	0	0.0	0.0000	6	100.0	0.0029	0	0.0	0.0000	13.0
	Chloroflexi	2	5.1	0.0021	0	0.0	0.0000	0	0.0	0.0000	4.3
	Unknown/unclassified	5	11.5	0.0066	0	0.0	0.0000	0	0.0	0.0000	9.7
	∑	39	100.0	0.0818	6	100.0	0.0029	1	100.0	0.0060	100.0
110m	Alphaproteobacteria	75	31.3	0.0516	0	0.0	0.0000	9	70.4	0.0042	29.6
	Betaproteobacteria	11	4.7	0.0118	0	0.0	0.0000	1	9.4	0.0030	4.4
	Gammaproteobacteria	30	12.5	0.0267	0	0.0	0.0000	1	11.8	0.0005	11.2
	Deltaproteobacteria	2	0.8	0.0074	0	0.0	0.0000	1	8.3	0.0023	1.1
	Archaea	31	12.9	0.0046	0	0.0	0.0000	0	0.0	0.0000	11.0
	Chlamydiae/Verrucom.	59	24.3	0.0309	0	0.0	0.0000	0	0.0	0.0000	20.7
	Gemmatimonadetes	1	0.4	0.0016	0	0.0	0.0000	0	0.0	0.0000	0.4
	Planctomycetes	1	0.4	0.0005	30	100.0	0.0102	0	0.0	0.0000	11.0
	Aquificae	21	8.7	0.0019	0	0.0	0.0000	0	0.0	0.0000	7.4
	Deinococcus-Thermus	1	0.4	0.0005	0	0.0	0.0000	0	0.0	0.0000	0.4
	Unknown/unclassified	9	3.5	0.0139	0	0.0	0.0000	0	0.0	0.0000	3.0
	∑	241	100.0	0.1513	30	100.0	0.0102	12	100.0	0.0100	100.0
200m	Alphaproteobacteria	202	46.8	0.1500	0	0.0	0.0000	2	24.4	0.0050	45.5
	Betaproteobacteria	57	13.2	0.0233	0	0.0	0.0000	4	51.2	0.0007	13.6
	Gammaproteobacteria	98	22.6	0.0463	0	0.0	0.0000	1	12.2	0.0022	22.0
	Deltaproteobacteria	4	0.8	0.0040	0	0.0	0.0000	0	0.0	0.0000	0.8
	Cyanobacteria	1	0.2	0.0004	0	0.0	0.0000	0	0.0	0.0000	0.2
	Chlamydiae/Verrucom.	24	5.6	0.0223	0	0.0	0.0000	0	0.0	0.0000	5.3
	Bacteriodetes/Chlorobi	2	0.5	0.0009	0	0.0	0.0000	0	0.0	0.0000	0.4
	Planctomycetes	6	1.3	0.0038	9	100.0	0.0149	1	12.2	0.0098	3.5
	Aquificae	18	4.2	0.0075	0	0.0	0.0000	0	0.0	0.0000	4.0
	Chloroflexi	4	0.8	0.0026	0	0.0	0.0000	0	0.0	0.0000	0.8
	Deinococcus-Thermus	1	0.1	0.0007	0	0.0	0.0000	0	0.0	0.0000	0.1
	Deferribacteres	1	0.2	0.0004	0	0.0	0.0000	0	0.0	0.0000	0.2
	Unknown/unclassified	16	3.7	0.0082	0	0.0	0.0000	0	0.0	0.0000	3.6
	∑	432	100.0	0.2704	9	100.0	0.0149	8	100.0	0.0178	100.0
